# Whole exome sequencing of FFPE samples—expanding the horizon of forensic molecular autopsies

**DOI:** 10.1007/s00414-022-02906-x

**Published:** 2022-11-08

**Authors:** Emma Adolfsson, Daniel Kling, Cecilia Gunnarsson, Jon Jonasson, Henrik Gréen, Anna Gréen

**Affiliations:** 1 Department of Laboratory Medicine, Faculty of Medicine and Health, Örebro University, Örebro, Sweden; 2 Department of Forensic Genetics and Forensic Toxicology, National Board of Forensic Medicine, Linköping, Sweden; 3 Department of Clinical Genetics and Department of Biomedical and Clinical Sciences, Linköping University, Linköping, Sweden; 4 Centre for Rare Diseases in South East Region of Sweden, Linköping University, Linköping, Sweden; 5 Division of Clinical Chemistry and Pharmacology, Department of Biomedical and Clinical Sciences, Linköping University, Linköping, Sweden

**Keywords:** WES, FFPE, SCD, Molecular autopsy, Arrhythmia, Cardiomyopathy

## Abstract

Forensic molecular autopsies have emerged as a tool for medical examiners to establish the cause of death. It is particularly useful in sudden unexplained deaths where the cause of death cannot be determined with a regular medical autopsy. We provide the first study of exome data from formalin-fixed paraffin-embedded samples (FFPE) paired with data from high-quality blood samples in forensic applications. The approach allows exploration of the potential to use FFPE samples for molecular autopsies and identify variants in extensive exome data. We leverage the high uniformity of the hybridization capture approach provided by Twist Bioscience to target the complete exome and sequence the libraries on a NextSeq 550. Our findings suggest that exome sequencing is feasible for 24 out of a total of 35 included FFPE samples. When successful, the coverage across the exome is comparatively high (> 90% covered to 20X) and uniform (fold80 below 1.5). Detailed variant comparisons for matched FFPE and blood samples show high concordance with few false variants (positive predictive value of 0.98 and a sensitivity of 0.97) with no distinct FFPE artefacts. Ultimately, we apply carefully constructed forensic gene panels in a stepwise manner to find genetic variants associated with the clinical phenotype and with relevance to the sudden unexplained death.

**Supplementary Information:**

The online version contains supplementary material available at 10.1007/s00414-022-02906-x.

## Introduction

Sudden unexplained deaths (SUDS) are often tragic, shocking, and devastating, especially when occurring in young people. A SUDS is defined as when a person over the age of 1 year dies suddenly and is often caused by sudden cardiac death (SCD). The term SCD is often used where a deceased person has died within 1 h of symptom onset, where the probable cause of symptoms was of cardiac origin, or where a person has died unexpectedly within 24 h after being seen alive and in a healthy condition [1, 2]. The incidence of SCD is difficult to determine and varies with age of the investigated population and the definition used for SCD in the study, but have been reported to be as low as 1.3 up to ≥ 100 cases per 100,000 individuals [3–5]. In Sweden, Wisten and Statin report an incidence rate of 0.93 per 100,000 per year in 15–35-year-olds [6]. The causes of SCD are distributed differently between different age groups [2, 7] where the proportion of coronary artery disease (CAD) related cases increases with age. The known causes of SCD can broadly be divided into structural and non-structural causes, see Table 1 [5, 7–9].

**Table 1:** List of known causes of SCD, based on Kumar et al. [5], Gray et al. [5], Isbister et al. [7], Musurunu et al. [61], and with information subtracted from OMIM [64]. The list includes, but is not limited to, structural and non-structural diseases

Structural diseases	Non-structural diseases
Arrhythmogenic right ventricular cardiomyopathy (ARVC)	Atrial fibrillation
Aortic disease	Catecholaminergic polymorphic ventricular tachycardia (CPVT)
Dilated cardiomyopathy (DCM)	Early repolarization syndrome (ERS)
Hypercholesterolemia—Coronary Artery Disease (CAD)	Idiopathic ventricular fibrillation
Hypertrophic cardiomyopathy (HCM)	Progressive cardiac conduction disease (PCCD)
Left Ventricular Non-Compaction Cardiomyopathy (LVNC)	Short QT syndrome (SQTS)
Muscular dystrophies	Ventricular tachycardia
RASopathy syndromes and Noonan disorders	Wolff-Parkinson-White Syndrome (WPW)
Infiltrative cardiomyopathies—Cardiac amyloidosis, Fabry disease, Hemokromatosis, Friedreich ataxia, Senger syndrome	Long QT syndrome (LQTS)—Timothy syndrome, Andersen-Tawil syndrome, Jervell and Lange-Nielsen syndrome (JLNS)
Other cardiomyopathies	

Heritability of SCD is pronounced but is again different for different age groups and depends on the causative disease [10]. The inheritance pattern can be complex, for instance with CAD, or autosomal dominant as for Long QT syndrome (LQTS) [10], but other inheritance patterns have also been observed. In cases involving young individuals (1–35 years of age), the heritability is more pronounced, and often autosomal dominant, but with incomplete penetrance and variable expression [11]. The main cause of SCD in this age group is inherited cardiac disease where genetic testing and family follow-up and investigation are recommended to prevent further cardiac-related deaths in the family [2]. However, in many of these cases, a SCD might be the first sign of the disease leaving the initial diagnosis to the clinical or forensic pathologist.

Forensic molecular autopsy, i.e. post-mortem genetic testing, is considered an important tool to uncover a likely or plausible cause of death, especially in SCD, and has been recommended by several instances with family follow-up and investigation to prevent further deaths in the family [1, 2, 9, 12, 13]. Genetic testing is particularly useful in autopsy-negative cases, which are often due to an underlying inherited arrhythmogenic cardiac disease. Many forensic laboratories have established molecular autopsies in their routine, but still genetic testing is warranted in older cases where high-quality material suitable for genetic analysis is no longer available. Ever so often, only formalin-fixed paraffin-embedded (FFPE) material is stored after autopsy, because it is the specimen of choice for histopathological diagnosis and it is easily kept at room temperature. However, extracting DNA suitable for massive parallel sequencing from FFPE is difficult since nucleic acids are sheared into fragments and formalin causes crosslinks between DNA, proteins and histones, as well as deamination of cytosine bases and the creation of abasic sites [14–17]. The use of post-mortem samples is difficult since the bodies might have degraded prior to sampling. Genetic testing in forensic applications has therefore mostly been limited to analysis of specific genes or smaller gene panels using molecular methods that works with fragmented FFPE material. Whole exome sequencing (WES) from FFPE has been advised to be used with caution as evidence indicates the presence of formalin-induced artefacts [18, 19]. Using new techniques that can overcome these obstacles and ensure the quality of WES from FFPE samples would be beneficial in a molecular autopsy setting. WES benefits from including all ~ 22,000 known protein-coding genes, which constitute 3% of the entire genome while containing ~ 89% of the pathogenic variants associated with genetic diseases [20]. The present study was undertaken with the aim to enable reliable WES of FFPE material for genetic analysis by building on a hybridisation based chemistry from Twist Bioscience, using a modified library preparation protocol with inclusion of unique molecular identifiers (UMI). The accuracy and precision of WES FFPE is investigated by using paired samples with a comparison of variants across the whole exome.

Evaluation of complete exome sequencing data is cumbersome, in particular in the last step of the bioinformatic approach whereby each detected variant has to be assessed for pathogenicity. A common approach to alleviate the burden with extensive sequencing data is to apply a filter where a smaller subset of genes is targeted. Each variant detected is assessed using established ACMG guidelines for pathogenicity [21]. If a causal variant cannot be found, an expanded gene panel is tested. The selected gene panel must be relevant to what is known about the circumstances at the time of death therefore the clinical description is vital when investigating a potential genetic cause of a presumed SCD [9]. The clinical value of WES FFPE was addressed by applying step-wise filters on the genetic variants from the FFPE samples in this study.

## Material and methods

The work described herein has been carried out in accordance with the code of ethics of the World Medical Association declaration of Helsinki. The research was approved by the regional ethics committee in Linköping, ethics permission numbers 2016–389-31 and 2020–06421. The individuals included in this study have been subject to previous routine forensic molecular autopsies. Since the individuals are deceased, obtaining formal consent is impossible. In accordance with the Ethics legislation and the Swedish transplantation law (SFS1995:831), a formal consent is not needed from relatives of the deceased, after ethical approval, since only already previously gathered material was used. In addition, all samples are pseudo-anonymized, making it impossible to trace any relatives of the deceased.

### Subject/specimen selection

Molecular autopsy case, *n* = 35, were selected from two cohorts where FFPE materials were available. In total, there were 23 men and 12 women, aged 0–72 years. The first cohort consisted of 15 forensic samples from two forensic genetics departments in Sweden, with paired DNA extracts from both FFPE and whole blood. These samples were used to validate the performance of the exome sequencing, since these data allowed comparisons and evaluation of potential artefacts induced by the state of the FFPE-derived samples. This cohort is referred to as the forensic samples. The second cohort consisted of 20 consecutive FFPE samples from clinical case work were FFPE material or FFPE DNA was the only available source of DNA. These were included to further evaluate the applicability of the protocol. These samples are referred to as clinical samples. The fixed-tissues were 1–23 years old; the time from death to tissue fixation, the fixation and embedding protocol, and storage conditions were unknown. For details, see Table 2. Furthermore, the Coriell platinum genome samples NA12877 and NA12878 (Coriell institute, Camden, USA) were included as high-quality controls to allow detailed comparisons using the reference platinum catalogue of contained SNVs in these samples [22].

**Table 2:** Demographics. All cases included in the project, *n* = 35. The table depicts sex, age when deceased, relevant autopsy findings, sample type received, and age of FFPE material

Sample ID	Cohort	Sex	Age when deceased (years)	Autopsy findings, relevant clinical features	Sample type	Age FFPE material (years)
Sample 1	Clinical	Male	69	SCD	Tissue block	8
Sample 2	Clinical	Female	59	HCM	Sections	4
Sample 3	Forensic	Male	1	Missing	Sections	3–4
Sample 4	Clinical	Male	24	Arrhythmia	Tissue block	22
Sample 5	Clinical	Male	34	Arrhythmia	DNA	2
Sample 6	Forensic	Male	19	Arrhythmia	Sections	2–3
Sample 7	Forensic	Male	1	SCD	Sections	1–2
Sample 8	Forensic	Male	23	Arrhythmia, palpations, and syncope	Sections	2–3
Sample 9	Clinical	Male	54	ARVC	Sections	11
Sample 10	Forensic	Male	38	SCD, tall statue, enlarged heart	Sections	2–3
Sample 11	Clinical	Male	50	SCD	DNA	5
Sample 12	Forensic	Male	32	Arrhythmia, possibly Brugada	Sections	2–3
Sample 13	Forensic	Male	21	Arrhythmia	Sections	2–3
Sample 14	Clinical	Male	55	Aorta dissection	Tissue block	1
Sample 15	Forensic	Female	1	SCD, lung infiltration	Sections	3–4
Sample 16	Clinical	Male	39	Ruptured aorta dissection	DNA	10
Sample 17	Forensic	Female	28	Long/short QT syndrome	Sections	1–2
Sample 18	Forensic	Female	49	SCD w/o structural findings	Sections	2–3
Sample 19	Forensic	Male	19	Arrhythmia, cardiac disarray patterns	Sections	3–4
Sample 20	Forensic	Male	30	Arrhythmia	Sections	2–3
Sample 21	Clinical	Male	48	Cardiomyopathy	Sections	1
Sample 22	Forensic	Female	62	ARVC	Sections	2–3
Sample 23	Clinical	Male	63	SCD/cardiomyopathy	Sections	3
Sample 24	Clinical	Male	55	HCM	Tissue block	1
Sample 25	Clinical	Female	53	SCD/cardiomyopathy	Tissue block	10
Sample 26	Clinical	Female	46	Sudden death	DNA	21
Sample 27	Clinical	Female	54	Dilated heart, lung emboli	Tissue block	11
Sample 28	Clinical	Male	0	DCM	Tissue block	23
Sample 29	Forensic	Female	24	Missing	Sections	2–3
Sample 30	Forensic	Female	36	Arrhythmia	Sections	2–3
Sample 31	Clinical	Male	69	TAAD	DNA	12
Sample 32	Clinical	Male	58	Possibly ARVC	DNA	15
Sample 33	Clinical	Female	59	HCM	Tissue block	21
Sample 34	Clinical	Female	72	SCD/cardiomyopathy	Tissue block	5
Sample 35	Clinical	Male	Missing	SCD/cardiomyopathy	Tissue block	Missing

Abbreviations: *SCD* sudden cardiac death, *HCM* hypertrophic cardiomyopathy, *ARVC* arrhythmogenic right ventricular cardiomyopathy, *TAAD* thoracic aortic aneurysm and dissection, *DCM* dilated cardiomyopathy

### Exome sequencing and bioinformatics

#### DNA extraction

From tissue blocks and/or frozen tissue sections, DNA was extracted using Magration System MagLEAD 12gc (Precision System Science Co., Ltd, Tokyo, Japan) with the FFPE DNA Purification Kit (ExScale Biospecimen Solutions, Uppsala, Sweden) following the manufacturer’s instructions using 3 × 10 µM tissue sections. If unsuccessful, repeated extraction attempts were done at least twice before resigning.

For the blood validation samples, *n* = 14, DNA had been extracted in conjunction with the molecular autopsy using the NorDiag Arrow procedures (NorDiag ADA, Oslo, Norway) and stored at − 80° C until the next steps. For a single sample, extraction was done using tissue (muscle) and the tissue protocol in the DNeasy Blood & Tissue Kits (Qiagen, Germany).

#### Quality control prior to library preparation

DNA concentration was determined using Invitrogen™ Qubit™ 2.0 Fluorometer using 1 × Qubit™ dsDNA HS Assay Kit (Invitrogen, Carlsbad, USA) following the manufacturer’s instructions.

For FFPE DNA, the quality and extent of fragmentation were analysed on 4200 Tapestation System (Agilent, Santa Clara, USA) using Genomic DNA Screen Tape following manufacturer´s instructions to obtain average fragment length (bp) and DNA Integrity Number (DIN)-score. The amplification potential was analysed using TruSeq FFPE DNA Library Prep QC Kit (Illumina, San Diego, USA) as per the manufacturer’s instructions to obtain qPCR scores, which describe the amplification potential.

#### Library preparation

The Twist protocol adopted for FFPE samples using UMIs and Westburg library preparation w/wo enzymatic fragmentation steps has previously been validated for SCD using the cardio diagnostic gene panel CDGP. In short, depending on the degree of fragmentation, the FFPE material was either fragmented further or just end-repaired prior to library preparation where UMI probes were incorporated, followed by pooling and hybridization with Twist ds DNA core exome probes with Twist Human Ref Seq spiked-in [23].

FFPE samples with average fragment length > 1000 bp and/or DIN-score > 3, *n* = 21, were selected for library preparation using Westburg NGS DNA Library Prep Kit (Westburg, Leusden, the Netherlands). Briefly, 250 ng of FFPE-DNA were enzymatically fragmented at 32° C for 4 min, followed by DNA end-repair and dA-tailing at 65° C for 30 min. Libraries from Coriell samples NA12877/NA12878 were prepared in the same manner using 50 ng input.

FFPE samples with average fragment length < 1000 bp and/or DIN-score < 3, n = 10, were selected for library preparation using Westburg NoFrag Library Prep Kit. No further fragmentation was done; instead, 250 ng of FFPE-DNA was only end-repaired and dA-tailed by incubating the samples for 30 min at 20° C, followed by 30 min at 65° C.

Duplex adapters containing UMIs (xGEN Duplex Seq Adapters, Integrated DNA Technologies, Inc., Coralville, USA) were ligated to the fragments by incubating at 20° C for 30 min. Ligated fragments were amplified in a pre-hybridization PCR (10–12 cycles) using KAPA HiFi HotStart Ready Mix (KAPA Biosystems, Wilmington, USA) and IDT duplex indexing primers (Integrated DNA Technologies, Inc., Coralville, USA), thereby adding 8 nt barcodes to each sample. Individual libraries were validated by measuring DNA concentration and by analyzing fragment length as described above. Successful library target values are > 80 ng/µl with an average fragment length of 375–425 bp. If unsuccessful, attempts to generate libraries were repeated at least twice.

Exome pools were generated using Twist Human Core Exome Multiplex Hybridisation Kit (Twist Bioscience, San Francisco, United States of America) in a modified protocol. Briefly, eight individual libraries (1500 ng of DNA) were pooled. The pool was hybridized at 70° C for 16 h using Twist Human Core Exome Kit and Twist Human RefSeq Panel. The exome library was amplified for 10 PCR-cycles using KAPA HiFi HotStart Ready Mix (Roche, Basel Switzerland), and xGEN Library Amplification Primer (xGEN Duplex Seq Adapters, Integrated DNA Technologies Inc., Coralville, USA). Pools were validated as above. Target values after whole exome library preparation were DNA concentration > 15 ng/µl, average fragment length 375–425 bp.

The matching DNA of the blood validation samples, as well as Coriell samples NA12877/NA12878, was prepared using Twist library preparation protocol as per the manufacturer’s instructions, with small adaptations. Briefly, 50 ng of DNA was enzymatically fragmented at + 32 C for 9 min, followed by end-repair and dA-tailing. Twist universal adapters were ligated to the fragments by incubation at + 20 C for 15 min. Twist 10 bp dual-indexed primers were added to each sample during 8 cycles of PCR. DNA concentration and fragment length were determined for FFPE samples. Successful library target values were > 80 ng/µl, with an average fragment length of 375–425 bp.

Exome pools were generated using the Twist Target Enrichment Protocol (Twist Bioscience, San Francisco, USA). In short, seven individual libraries were pooled to a total DNA pool mass of 1500 ng. The pool was hybridized at + 70° C for 16 h. After hybridization, the exome library was amplified for 8 cycles using KAPA HiFi HotStart Ready Mix and Twist amplification primers. DNA concentration and fragment length were determined as in the FFPE protocol, with the same target values as for the exome FFPE pool.

#### Sequencing

Sequencing was performed on a NextSeq 550 (Illumina, San Diego, USA) following the manufacturer’s instructions. Eight FFPE samples at a time were sequenced on a high-output kit (300 cycles) using ~ 1 pM as input. Repeated library generation and sequencing were performed in cases of unsuccessful outcome with at least two attempts per clinical FFPE case. Matching blood validation samples were sequenced on a high output kit (300 cycles), with 14 samples in a pool using ~ 1 pM as input.

#### Bioinformatic pipeline

The bcl files from the FFPE samples were converted into fastq files using bcl2fastq [24], with simultaneous removal of adapters and UMI, and inclusion of UMI into fastq headers. All reads were trimmed by 5 bp in the 3’ end to avoid UMI remnants. Burrows-Wheeler algorithm (bwa-mem 0.7.17) was used to align trimmed sequences to the reference genome. Specifically, the most recent human genome build 38 (GRCh38) was used throughout all experiments unless otherwise stated. A target BED file was created based on the file supplied by the manufacturer (https://www.twistbioscience.com/resources/bed-file/ngs-human-core-exome-panel-bed-files) with an additional 10 bp flanking each exon. Variants were called using GATK 3.8 [25], SAMtools 1.9 [26], and freebayes 1.10.46 [27] simultaneously. Ensemble multivcf files were constructed including variants called by at least two of the approaches. Sample-specific files were obtained from the multisample vcf using vcftools or alternatively R (version 4.1.0). CLC Genomic Workbench 22.0 (Qiagen) was used for targeted sequencing controls.

Blood samples were processed as described for the FFPE samples with the exception of the UMI removal step, whereas blood samples did not contain UMI sequences.

### Comparison and evaluation of quality controls, variants, and usefulness

Evaluation of data is performed using four different steps. First, the feasibility of extracting and sequencing DNA from FFPE material is assessed. Second, sequencing metrics, e.g. % ≥ 20X and uniformity, is summarized. Third, concordance for matched FFPE/blood samples is conducted. Fourth, an assessment of the clinical value to resolve cases of sudden unexplained deaths. Below, each step is described in detail. All data analysis is performed using custom scripts in R version 4.1.0 [28].

#### Feasibility of the WES FFPE protocol

To investigate the feasibility of the WES FFPE protocol, all 35 FFPE cases were included. Focusing on quality measurements prior to analysis, the correlation between the quality measurements of extracted DNA (average fragment length, DNA integrity, and amplification potential) and obtained libraries, as well as sample sequencing quality, were investigated to evaluate if and to what extent the quality control parameters could predict successful sequencing of the 35 samples.

#### Performance of the WES FFPE protocol

Sequencing metrics were obtained using CLC Genomic Workbench version 22.0. (https://digitalinsights.qiagen.com). Specifically, the “QC for Targeted Sequencing” tool was used to obtain base-specific coverage values and multi-QC [29] was used to obtain summary statistics (e.g. mapped reads, insert size) for each sample separately.

#### Concordance of the WES FFPE protocol to paired blood samples

To assess the accuracy, a two-step validation procedure was used. First, the variant calling for two Coriell samples (NA12877 and NA12878) was compared to platinum genome files. Briefly, platinum genome vcf: s were retrieved from https://github.com/Illumina/PlatinumGenomes. The files were processed in bedtools version 2.27.1 [30] to find overlapping and confident regions with the target BED file, as previously described. As eluded to in the “Subject/specimen selection" section, the Coriell samples were subject to the FFPE and blood protocol separately to investigate potential effects from the protocol itself, generating two vcf: s for each Coriell sample. Variants detected in the platinum vcf were defined as true and consequently overlapping variants in the FFPE or blood sample as true positives (TP) and variants not detected in our samples as false negatives (FN). Extra variants in the FFPE and blood samples were defined as false positives (FP). Sensitivity was calculated as TP/ (TP + FN) and positive predictive value (PPV) as TP/ (TP + FP).

Secondly, a variant validation was performed using the successfully sequenced matched FFPE and blood samples (*n* = 13). In the first raw comparison, variants called in blood were defined as true, although low coverage might be the explanation for variants called in FFPE but not in blood and vice versa. Consequently, variants not detected in FFPE were defined as false negatives (FN) and conversely, variants not detected in blood were defined as false positives (FP).

#### Clinical value of the WES FFPE protocol

To determine if a genetic cause could be found in cases of suspected SCD, variants from sequencing the FFPE material were annotated and assessed using ACMG guidelines. A tier-based approach was used where gene panels were carefully designed for each step in the procedure. In the first tier, a previously published cardio diagnostic gene panel (CDGP) was used. The panel encompasses 84 genes correlated to cardiac diseases and has been used extensively for cardiomyopathies, arrhythmias, and SCD in both forensic and clinical genetics [23, 31]. In the second tier, a larger sudden death gene panel (SDGP) was constructed to complement and expand the CGDP. PanelApp [32], ClinGen [33], and extensive literature searches were used to add (a) missing genes related to classic genetic cardiac diseases like hypertrophic cardiomyopathy (HCM) and long QT syndrome (LQTS), (b) genes involved in not yet high-level evidence genetic heart diseases, (c) genes involved in diseases with a phenotype of cardiac disease such as Noonan’s disorder. In total, the larger sudden death gene panel contains 166 genes. Furthermore, an aorta panel containing 27 genes was constructed based on ClinGen [33] and Pinard [34]. For detailed gene panel contents, see Supplementary Tables 1–3.

Raw vcf files were filtered and analysed using QCI Interpret (Qiagen). Variants were filtered using an allele frequency cutoff equal to 5% in gnomAD (overall variant allele frequency) [35]. Further filtration was subsequently done in QCI Interpret, retaining variants with allele frequencies < 1% in gnomAD as well as established pathogenic variants with a frequency above 1%. The filtering strategy was subsequently performed on a case-to-case basis according to written referrals from pathologists or clinical geneticists, or alternatively according to autopsy findings.

The referrals were divided into the following categories with increasing complexity:

1. *Investigation of a familial variant*
  A specific variant had previously been detected in relatives of the patient and only the relevant genomic position was examined to establish the presence of the familial variant.
2. *Investigation of a specified gene*
  Referrals for investigation of a certain gene/genes. Filtering was performed to only retain variants in the indicated gene/genes.
3. *Investigation of a well-described genetic cardiac disease or condition*
  Requests for genetic analysis of a certain disease, for example, thoracic aortic aneurysm and dissection (TAAD). In these cases, analysis using a targeted panel (for example, the aorta panel described previously) was performed.
4. *Referral for cardiomyopathy and/or SCD in general*
  The two-tier approach, described above, was applied were a smaller panel (CDGP) was first applied and subsequently an expanded panel (SDGP) to find genetic variants linked to SCD.

After filtering, all remaining variants were assessed for pathogenicity using ACMG guidelines [21]. All variants were evaluated using QCI-I (Qiagen) and Alamut (SophiaGenetics, Boston, USA) and manually inspected using IGV [36]

## Results

### Feasibility of the WES FFPE protocol

DNA in sufficient quantity (yield > 250 ng) was extracted from 23 FFPE samples, and in sub-sufficient quantity (yield 50–249 ng) from 5 samples. From a single FFPE sample, DNA extraction failed. From the received, already extracted, DNA samples (*n* = 6), a single sample was excluded after quality control as DNA concentration was insufficient to continue with library preparation. In total, 33 FFPE samples were subject to library preparation. Pre-library preparation quality control of the extracted DNA revealed a moderate to a high degree of fragmentation (median 1614 bp, range 266–3296), low DIN-scores (median 3.1, range 1.2–6.1), and a wide range of qPCR-values (median 5.2, range 0.8–14.8), see Supplementary Table 4.

**Fig. 1:**
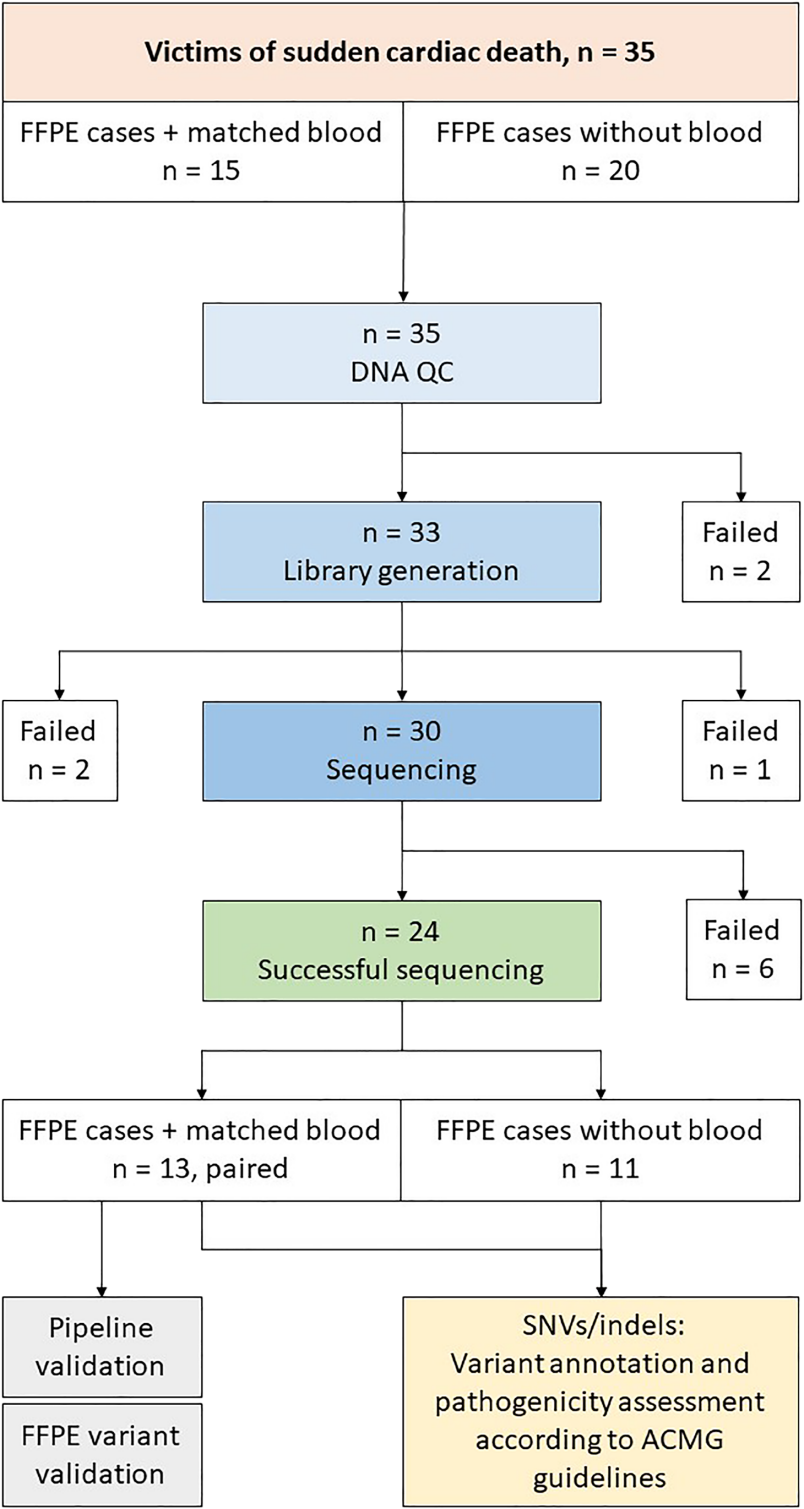
Flowchart illustrating the path from sample extraction to variant calling. In total, WES FFPE was attempted for 35 cases, 15 of these had matching blood samples and are considered validation forensic samples. The other samples were consequentially received clinical requests for genetic testing where only FFPE material remained. After DNA quality control, 33 cases remained. Library generation was therefore initiated for 33 cases. Libraries of sufficient quality were generated from 30 cases, and three cases failed library generation despite several attempts. Whole exome sequencing was performed for the 30 cases, in batches of eight, and sequencing was successful for 24 of these, 13 of these were the validation forensic samples. These samples were used for pipeline validation and variant comparisons. All 24 successfully sequenced samples were analysed with variant annotation and pathogenicity assessment according to ACMG guidelines [21]

Libraries could be generated from 30 of the 33 available samples. Target values for Twist libraries are 375–425 bp, but most of the libraries from FFPE samples were shorter, with an average length f 267 bp, range 152–468 bp. Samples that failed library prep had qPCR > 8, DIN-value < 2 and DNA fragment length between 300 and 1000 bp. Sequencing was successful for 24 of the 30 samples and a flow chart of the study can be seen in Fig. 1.

### Performance of the WES FFPE protocol

Initial analysis indicated that FFPE samples had to be pooled in batches of 8 on a high output kit (theoretical yield of 800 M paired-end reads) on a NextSeq 550 instrument to ensure sufficient coverage across all samples, whereas high-quality blood samples were pooled in batches of 14 samples on a high output kit, which in theory yields close to twice the number of raw sequence for the FFPE samples. Table 3 contains a summary of the samples sequenced in this study where the amount of average mapped reads is twice as high for FFPE samples owing to the sequencing setup previously mentioned and the variation in the amount of mapped reads is considerable for FFPE samples. It is noteworthy that the duplication rate is extremely low for the blood samples, as expected for high-quantity input, but also low for the FFPE samples, an average of 8%.

**Fig. 2:**
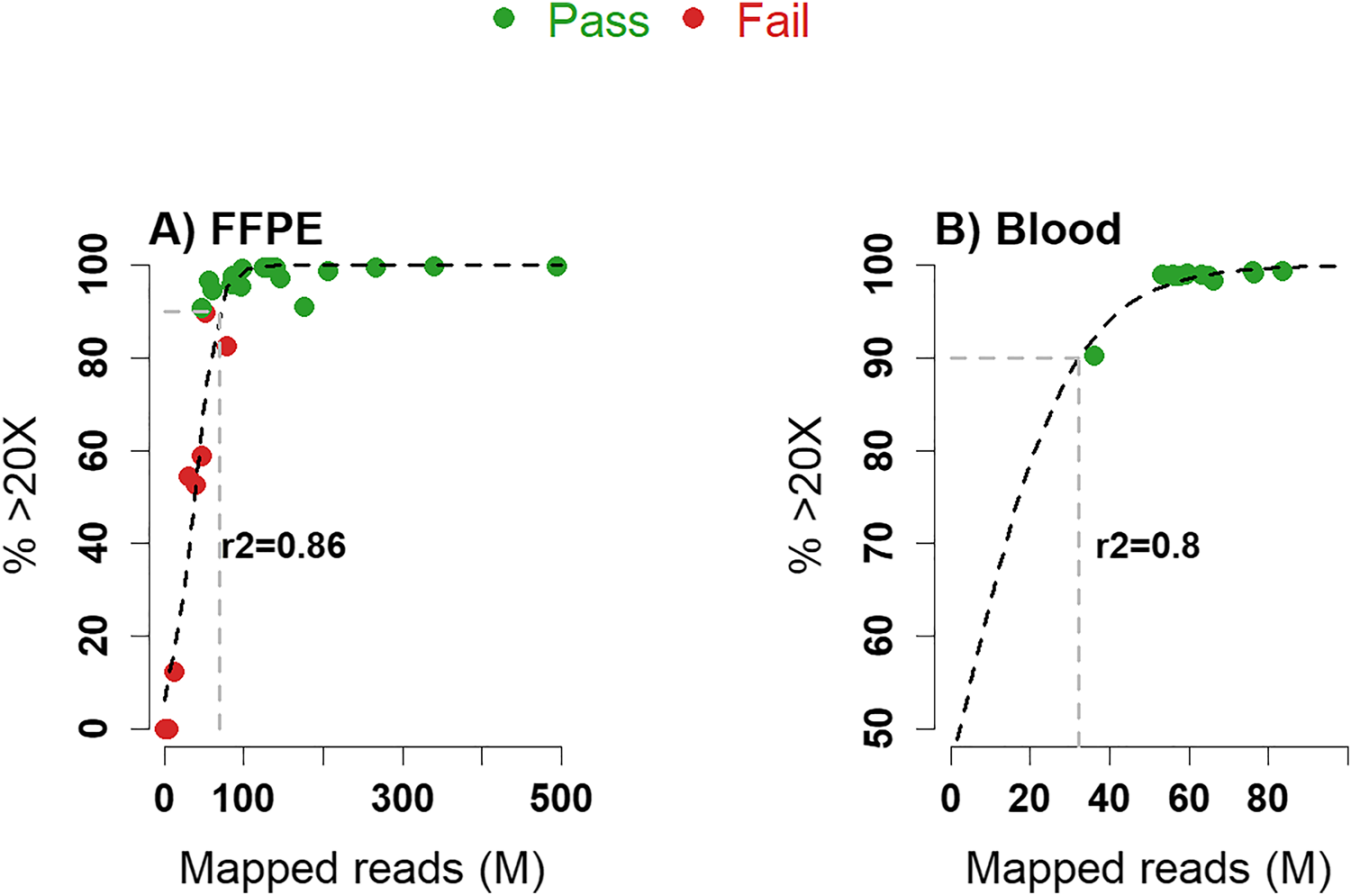
Correlation between the number of mapped reads in millions (x-axis) and the percentage of targets covered to 20X. **A** FFPE samples and **B** blood samples. The *r*^2^ value is the goodness of fit for a logistic regression using a binomial distribution, also plotted in the graph as a dashed black line. Dashed grey lines illustrate the intersection between the amount of mapped reads and the point where 90% of the targets are covered to at least 20X. Note the difference in scale. Abbreviation: FFPE = formalin-fixed paraffin embedded

Successful sequencing was defined as when 90% of the targets were covered to at least 20X. Figure 2 correlates the percentage of targets covered to 20X to the number of mapped reads for each sample and illustrates that at least 50 M reads are required for a FFPE sample to exceed the 90% threshold. All blood samples exceeded this threshold, see Fig. 2B. The FFPE samples failing to exceed this threshold were investigated in further detail to scrutinize whether sufficient coverage was obtained in the subset of genes addressed in each case, see the “Concordance of WES FFPE protocol to paired blood samples” section.

**Fig. 3:**
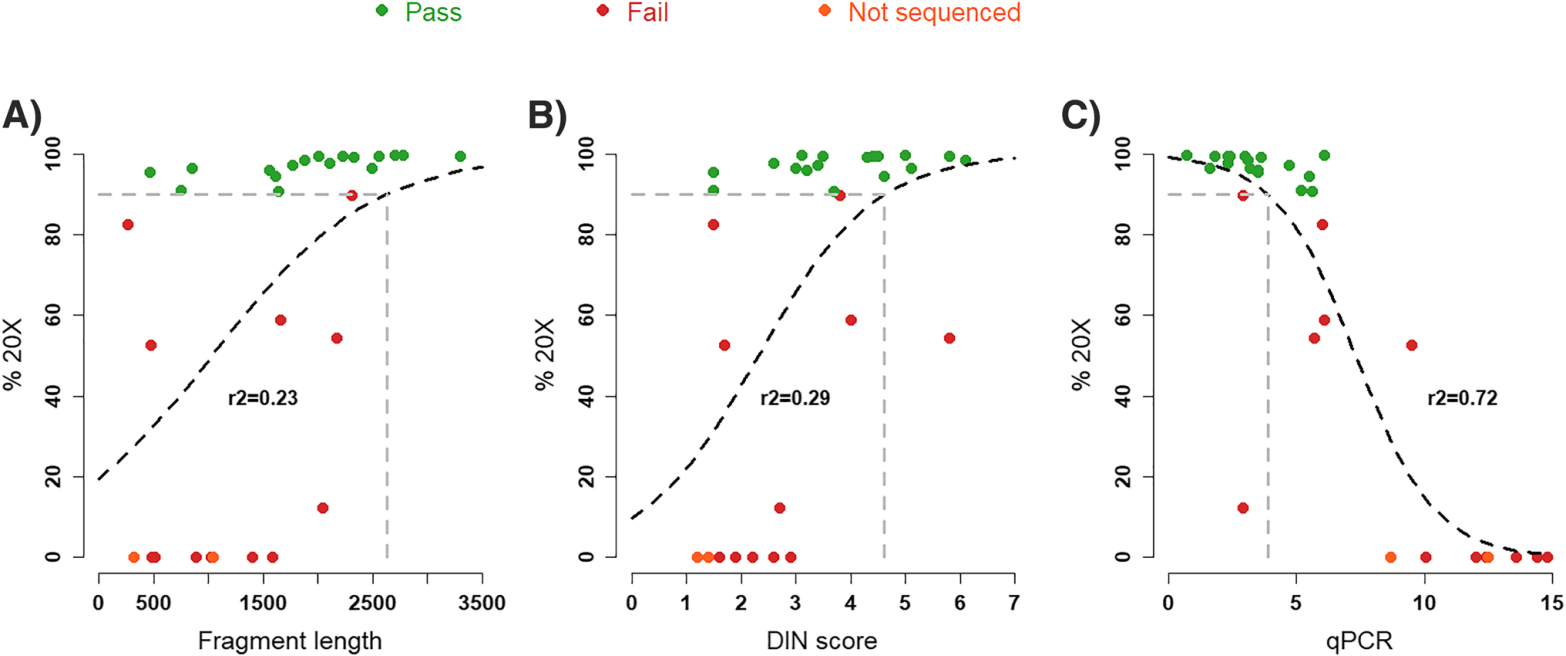
Correlation between three different quality metrics and the percentage of targets covered to 20X. **A** Average fragment length, **B** DIN score, and **C** qPCR value. The r2 value is the goodness of fit for a logistic regression with a quasibinomial fit in R, also plotted in the graph as a dashed black line. Dashed grey lines illustrate the intersection between the measures quality metric and the point where 90% of the targets are covered to at least 20X

Three pre-library quality metrics from 24 successfully sequenced FFPE samples were correlated to the percentage of targets covered to 20X. Figure 3 summarizes the results illustrating a fairly strong correlation (Spearman’s correlation coefficient =  − 0.79, *p* = 1.16E-7) between the qPCR value and the sequencing success whereas the fragment length and the DIN score have a lower, but still significant, correlation to a successful outcome (Spearman’s correlation coefficient = 0.62 and 0.68 respectively).

As illustrated in Fig. 2A and Supplementary Table 4, there is great variation in the number of mapped reads for the different FFPE samples, owing to the variation in input quality. As expected, high-quality samples (i.e. qPCR values < 10) obtain a greater number of raw reads while, in contrast, low-quality samples obtained a low number of reads. Supplementary Fig. 1 illustrates a weak correlation between the qPCR value and the fraction of obtained reads for individual sequencing batches (Spearman’s correlation test yields a negative correlation, similar to Fig. 3, with *p* values < 0.05 for three of the included batches while the fourth batch does not illustrate a significant correlation). It is therefore important to consider sample quality measures (e.g. qPCR value) before pooling samples, where samples of roughly equal quality will result in more successful and even sequencing across samples. Sequencing high-quality samples together with low-quality samples will otherwise result in a loss of reads for the low-quality samples, while high-quality samples will obtain an excess.

**Fig. 4:**
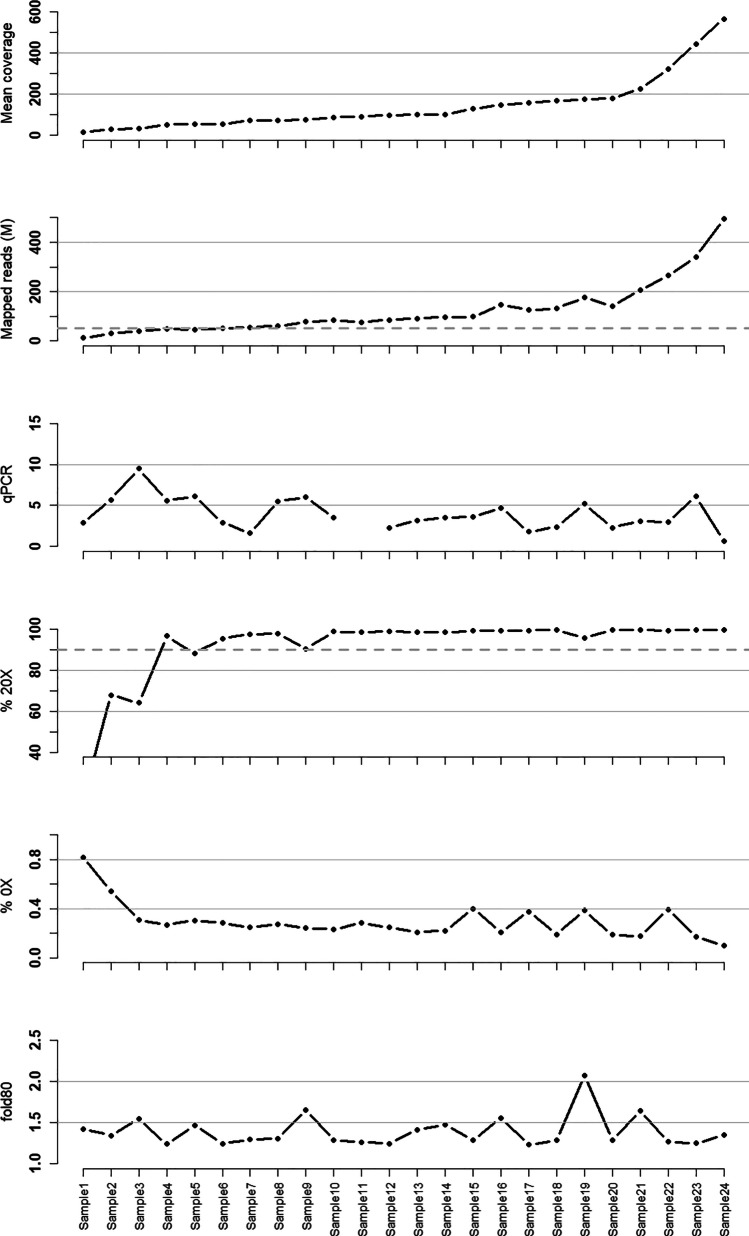
Sequencing metrics across the 24 FFPE samples with successful sequencing. From top-to-bottom, the qPCR value, mean coverage across all target bases, fraction of target bases covered to at least 20X, fraction of target bases covered to 0X and fold80 illustrating the uniformity of the data. Samples are sorted based on median coverage

Figure 4 summarizes sequencing metrics across the entire set of FFPE samples (*n* = 24) that yield sequence data, illustrating the high uniformity with fold80 base penalty measures [37] below 1.5 for the majority of samples, even in low-quality samples. Comparison between matched blood and FFPE showed uniformity of sequence across the high-quality blood samples (mapped reads) and within samples (fold80-based penalty), see Supplementary Fig. 2 for details.

To find regions with consistent low coverage across all samples, an approach whereby the coverage data for the FFPE samples (*n* = 24) were processed jointly was used for base positions where one of the following criteria was fulfilled:

1. At least 6 out of 24 FFPE samples (25%) had zero coverage (0X)
2. The maximum coverage across all samples was below 20X
3. The sum of the coverage across all 24 FFPE samples was less than 100.
4. The median coverage across all samples was below 20X.

Either of the above points would suggest problems with the design or mapping issues. Supplementary Fig. 3 illustrates a summary of the results, divided into chromosomes for visualization.

**Fig. 5:**
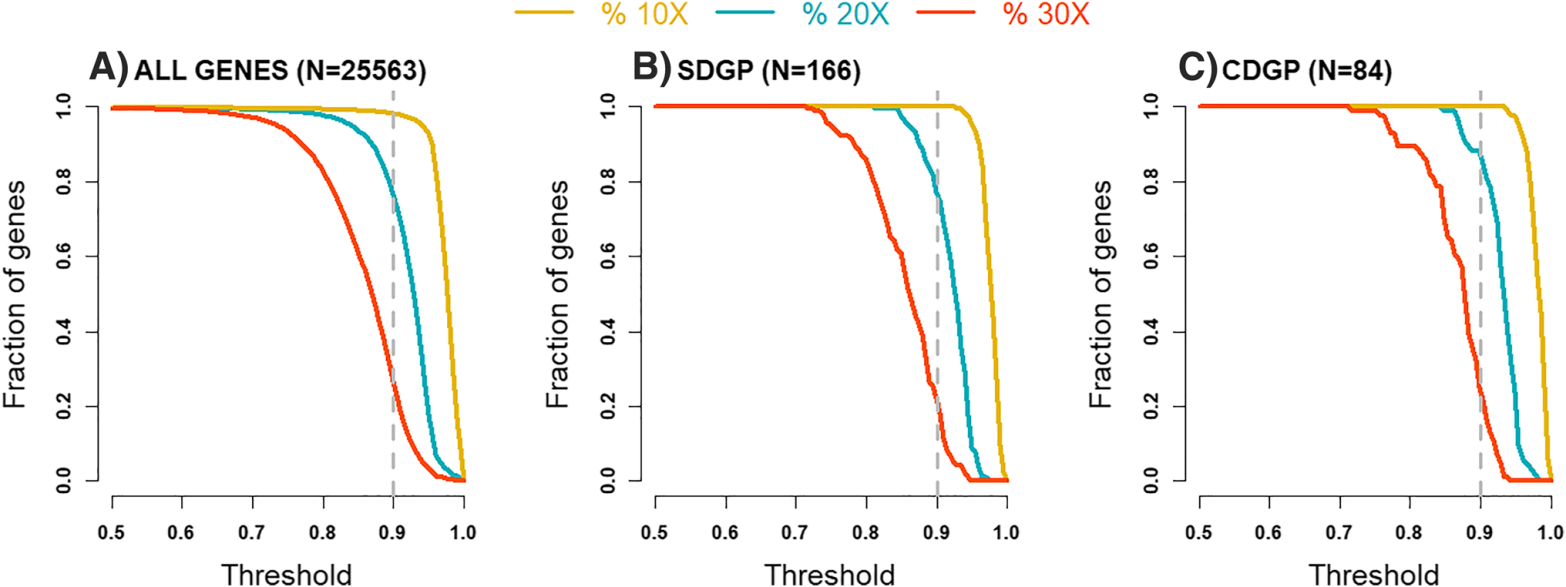
Fraction of genes covered to at least 10X, 20X, and 30X respectively. To consider a gene covered we use a sliding threshold (x-axis), where 0.5 indicates that at least 50% of the gene is covered to at least the specific coverage. **A** All genes are included from the exome (*n* = 255,563); **B** SDGP, Sudden Death Gene Panel, *n* = 166; and **C** CDGP, Cardio Diagnostic Gene Panel, *n* = 84. The dotted line corresponds to 90% of the gene covered

A separate analysis was performed where the coverage across all genes was summarized in the complete set of 24 FFPE samples. Figure 5 illustrates the results where a sliding threshold (x-axis) for the percentage of a gene being covered at a specific threshold in order for it to be considered covered is used.

### Concordance of the WES FFPE protocol

Table 3 shows the results from the variant comparison using the platinum genome samples (NA12877 and NA12878) with high PPV (> 0.99) and sensitivity (> 0.989) for both samples and protocols used (FFPE and standard). Initial analysis showed that several false positive/negative variants were located in problematic regions or regions with poor coverage (below 20X). For example, regions with tandem repeats, homopolymer content, NGS problematic regions, and regions with bad promoters. To reduce the false positives/negative rates, bedtools was used to find the intersect between stratification files downloaded from https://ftp-trace.ncbi.nlm.nih.gov/ReferenceSamples/giab/release/genome-stratifications/v3.0/GRCh38/ and our BED file, see Table 4 for a summary. Variants in these regions will not be used in downstream variant interpretation due to the high degree of complexity in terms of alignment and genotype calling. In addition to the stratification files applied in this study, further problematic regions can be accessed in the link above, e.g. regions enriched for segmental duplications or GC content, but where the intersect, and therefore reduction of the regions investigated, is large, ~ 2 Mb.

**Table 3:** Summary of variant comparisons for two Coriell samples (NA12877 and NA12878) and platinum genomes. Each Coriell sample has been analysed in parallel using the protocol for blood and for FFPE respectively

Parameters	NA12877	NA12878
Variants in Platinum	23,821	24,162
Variants in blood	24,064	24,346
Variants in FFPE	24,022	24,350
Overlap with blood	23,806	24,137
Overlap with FFPE	23,806	24,127
Overlap blood and FFPE	23,992	24,302
PPV blood	0.999	0.998
PPV FFPE	0.999	0.998
Sensitivity blood	0.989	0.991
Sensitivity FFPE	0.991	0.991

*FFPE* formalin-fixed paraffin-embedded, *PPV* positive predictive value

**Table 4:** Stratification files used in this study and the intersect with our target BED file. Note that the first row of the table (Confident region) includes regions whereas the remaining excludes regions

Stratification file	Size (Mb)	Percentage of total (%)
Confident regions	38.045	92.98
Tandem repeats and homopolymers	1.771	4.3
Bad promoters	0.017	0.4
Low mappability	2.193	5.36
NGS high stringency regions	1.855	4.53
Internal regions	0.073	0.17
All other difficult regions	0.544	1.33

**Fig. 6:**
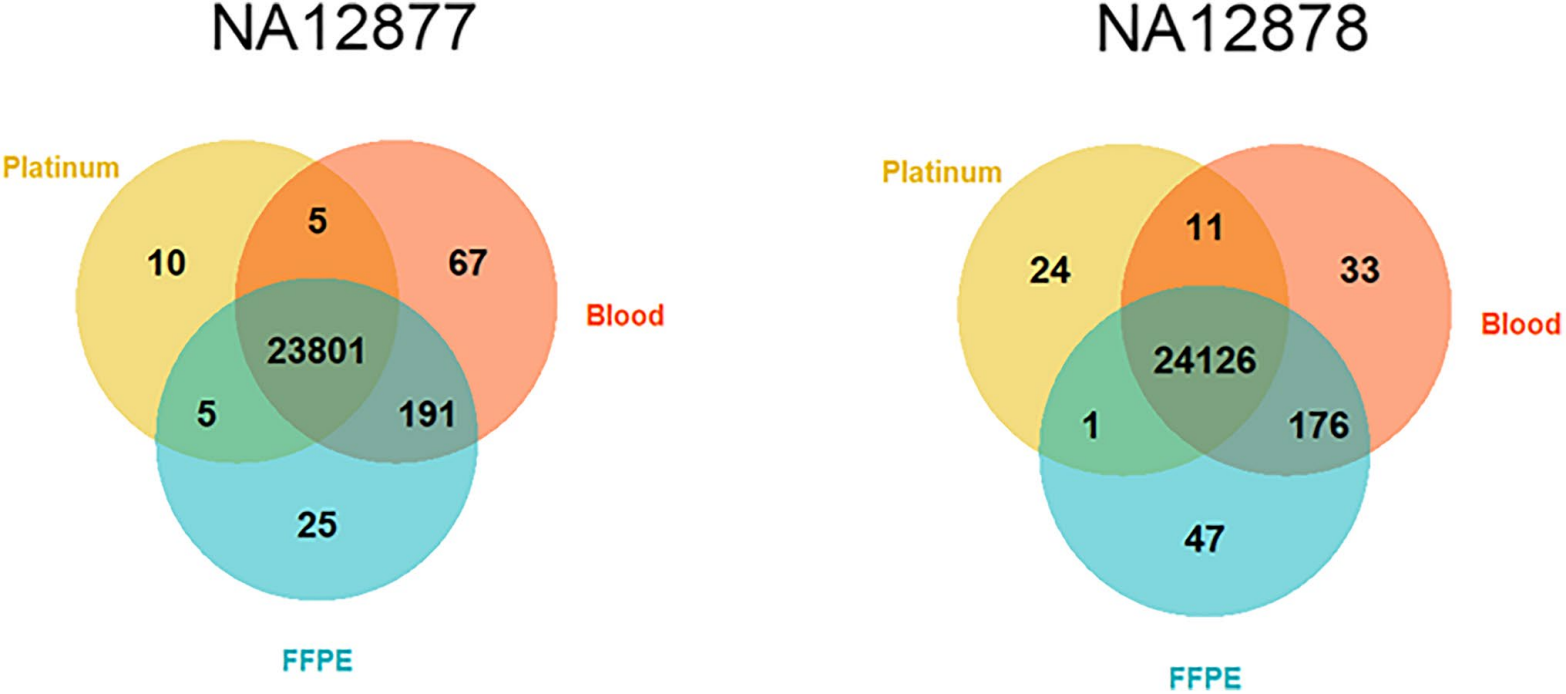
Venn diagrams illustrating a variant comparison between platinum genomes and the Coriell samples analysed in this study. The Coriell samples are sequenced twice, once using the protocol for high-quality blood samples and once with the protocol used in this study for FFPE samples. Numbers illustrate unique variants for each sample type as well as the overlap, where the area is not proportional to the number of variants in each set. FFPE formalin-fixed paraffin-embedded

Figure 6 and Table 4 summarize the results from the comparison of the platinum genomes (NA12877 and NA12878) and indicate high sensitivity (above 0.998% for all comparisons), i.e. variants in the platinum files are detected and called in our samples. In contrast, the positive predictive value is slightly lower, but still almost 0.99 in all samples, indicating a higher degree of false variants in our calling. The results in Table 4 further show similar results (PPV and sensitivity) for the Coriell samples analysed on the FFPE and the blood protocol indicating no or small effects from the different protocols. Closer investigation revealed that the false negative variants could be broadly categorized into (1) insertion/deletion or (2) located in regions with low variant frequency in our data, i.e. skewed allele balance. In the platinum genomes, a single caller (platypus) had in the majority of cases called these variants. The false positives, which greatly outnumber the false negatives (Fig. 6), are mostly located in regions with known problems, but not part of our stratification files, see Supplementary Table 5. In total, 102 − 129 false positive variants remain when the intersects with problematic regions are discarded. Of the remaining variants, a great number are located in regions enriched for variants, see Supplementary Fig. 4.

Table 5 summarized the raw variant comparison for the matched FFPE and blood samples, illustrating a comparatively high sensitivity (average of 0.98 across the samples) and PPV (average of 0.97 across the samples). To determine the cause of each of the non-overlapping variants a series of steps were performed. First, complete coverage data was generated for all samples across the entire target BED file (in total roughly 40.9 Mb). Next, the intersect between regions covered to 20X in the matched samples (FFPE and blood) was generated. Secondly and similar to the approach taken for the Coriell samples, the intersect with known problematic regions (see Table 3) and the non-overlapping variants were summarized. Thirdly, all non-overlapping variants were clustered into regions using an iterative approach in R. Briefly, using a sliding window of 100 bp, non-overlapping variants were merged into larger regions. This allowed the definition of an internal database of highly problematic regions enriched for non-overlapping variants, see for instance Supplementary Fig. 4.

**Table 5:** Variant comparison between matched blood/FFPE samples. The table summarizes the raw vcf file comparisons. Displayed as mean for 13 matched samples with variation in parentheses

Parameters	Mean (variation)
Variants in blood	33,569 (32,944–34,928)
Variants in FFPE	33,328 (32,255–34,871)
Overlapping variants	32,546 (31,456–34,175)
Not in FFPE (false negatives)	1023 (687–1984)
Not in blood (false positives)	784 (633–1061)
PPV	0.98 (0.97–0.98)
Sensitivity	0.97 (0.94–0.98)

*FFPE* formalin-fixed paraffin embedded, *PPV* positive predictive value

**Fig. 7:**
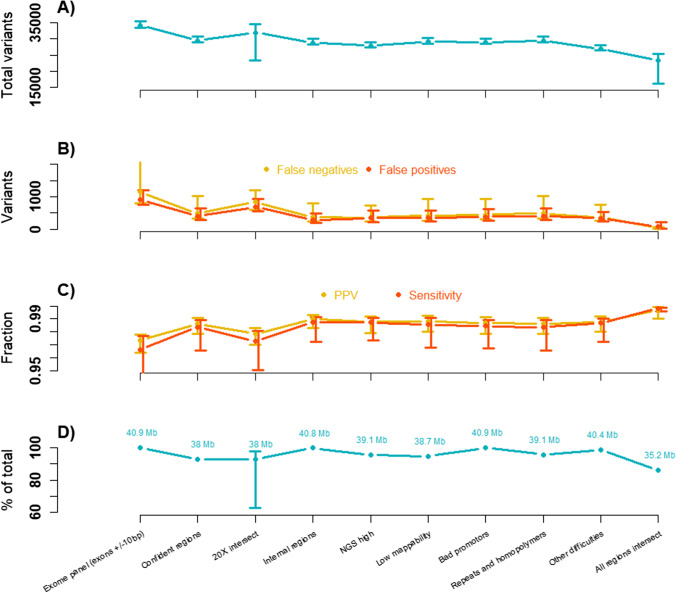
Summary of variant comparisons between matched FFPE and blood samples (*n* = 13) using different stratification files (x-axis). The exact contents of the regions on the x-axis are detailed in the main text. From top to bottom: **A** Total variants in each blood sample, **B** False positive and negative variants for each FFPE sample, **C** Positive predictive value (PPV) and sensitivity for each FFPE sample, **D** The size of each region (intersect with the target BED file). For **A**–**C**, the illustration contains the mean and the max/min for each region across the FFPE samples, and in **D,** the 20X intersect regions illustrate the mean and max/min for the FFPE samples

The results are summarized in Fig. 7 illustrating that while the removal of problematic regions (stratification) will reduce the size of the targets (from 40.9 Mb down to 35.2 Mb), very few false variants will be called (from an average of 906 to 69 false positives and an average of 1151 to 39 false negatives).

### Clinical value of the WES FFPE protocol

A molecular autopsy was done through variant interpretation in the 24 successfully sequenced SCD cases using WES FFPE data. As detailed in the “Material and methods” section, first all common variants with allele frequency < 1% in gnomAD were excluded, unless the variant is an established pathogenic variant in which case variants with higher frequencies could potentially be retained. Secondly, additional filtering was done based on phenotypic descriptions and/or autopsy findings. Table 6 summarizes clinically relevant findings and interpretations detailed below. Supplemental Table 5 includes the outcome of molecular autopsy for all FFPE samples.

**Table 6:** Clinical findings in the FFPE samples. The table includes samples with genetic variants clinically relevant to their phenotype. Table depicts analytical request, filtering strategy and genetic findings described with transcript, coding position, protein shift, zygosity, and pathogenicity according to ACMG guidelines. Finally, the variant is interpreted in the context of case clinical findings

Sample	Cohort	Request	Analysis strategy	Gene	Reference sequence	Variant DNA	Variant Protein	Zygosity	Hereditary pattern	ACMG classification	Interpretation in context of case clinical findings
Sample1	Clinical	SCD. Familial variant in DLL4	Familial variant in *DLL*4	*DLL4*	NM_019074.3	c.1096 T > G	p.(Cys366Glu)	Heterozygote	-	-	Familial variant confirmed
Sample2	Clinical	HCM	CDGP	*MYBPC3*	NM_000256.3	c.2490dupT	p.(His831fs*2)	Heterozygote	AD	Pathogenic	Likely cause of HCM
Sample4	Clinical	Analysis of *KCNQ1*	*KCNQ1*	*KCNQ1*	NM_000218.2	c.573_577del,	p.(Arg192Cysfs*91)	Heterozygote	AD	Pathogenic	Likely cause of SCD
Sample11	Clinical	SCD	SDGP	*CALM2*	NM_001743.6	c.356A > T	p.(Asp119Val)	Heterozygote	AD	Likely pathogenic	Possibly relevant for SCD
Sample12	Forensic	Arrhythmia, ERS, possibly Brugada	CDGP	*CACNB2*	NM_201590.3	c.1717C > T	p.(Arg573Cys)	Heterozygote	AD	VUS	Possibly relevant for SCD
Sample16	Clinical	Ruptured aorta dissection	Aorta panel	*FBN1*	NM_000138.4	c.1615C > T	p.(Arg539Trp)	Heterozygote	AD	VUS	Possibly relevant for ruptured aorta
Sample19	Forensic	Arrhythmia, cardiac disarray patterns	CDGP	*TNNC1*	NM_003280.3	c.435C > A	p.(Asp145Glu)	Heterozygote	AD	VUS	Possibly relevant for SCD
Sample24	Clinical	HCM	CDGP	*LDLR*	NM_000527.5	c.1246 C > T	p.(Arg416Trp)	Heterozygote	AD	Pathogenic	Pathogenic for FH, unclear relevance for HCM

Abbreviations: *AD* autosomal dominant, *AR* autosomal recessive, *ARVC* arrhythmogenic right ventricular cardiomyopathy, *CDGP* cardio diagnostic gene panel, *ERS* early repolarization syndrome, *FFPE* formalin-fixed paraffin-embedded, *FH* familial hypercholesterolemia, *HCM* hypertrophic cardiomyopathy, *LQTS* long QT syndrome, *SCD* sudden cardiac death, *SDGP* sudden death gene panel, *SQTS* short QT syndrome, *VUS* variant of uncertain significance

#### Investigation of a familial variant

Three cases were referred for investigating the presence of a specific familial variant. Two of the cases did not produce sequencing results. In the successful case, the familial variant was detected in *DLL4*, NM_019074.3: c.1096 T > G, despite low coverage (14.4% of bases covered above 20X in the exome) which allowed analysis of the presence of the specified variant, which cannot be assumed to be the cause of death. The case illustrates that even though overall exome coverage is low, local variant coverage could still be sufficient for a clinical answer.

#### Investigation of a specified gene

In one case, a referral was done for investigation of a certain gene (*KCNQ1*), due to clinical phenotype of the deceased. Despite that the sample had a suboptimal coverage (64.8% of the bases covered 20X in the exome) a pathogenic variant in *KCNQ1*, NM_000218.2: c.573_577del, was called, which is in agreement with the post-mortem findings of the case. Loss of function may result from protein truncation or non-sense mediated decay, which is a known disease mechanism for LQTS/Romano Ward syndrome. This variant has previously been observed multiple times in individuals with LQTS [38–40]. It has also been recognized as having a Swedish and Norwegian founder effect [40], but the QT-prolonging effect of heterozygous carriers is variable [41]. The variant is present in the European population in gnomAD at 0.003%. The same variant was also detected in a sister to the deceased.

#### Investigation of a well-described genetic cardiac disease with a specific gene panel for TAAD

Referral requests for genetic analyses of a certain disease were received for four cases, all for TAAD. Two samples were amplified successfully, while two samples could not be analysed due to poor sample quality. In one case a missense variant of unknown significance (VUS) in *FBN1,* NM_000138.4:c.1615C > T. This variant is not present in gnomAD and is located in a highly conserved region of fibrillin-1, where other pathogenic genetic variants causing TAAD and Marfan syndrome have been documented before. The variant may therefore be relevant for the patient’s disease (ruptured aorta).

#### Referral for cardiomyopathy/SCD in general

The CPDG (84 genes, listed in Supplementary Table 2) was used as a first step for analysing referrals for cardiomyopathy and/or suspected SCD. In one clinical case, a pathogenic frameshift variant was detected in *MYBPC3*, NM_000256.3:c.2490dupT, which was consistent with HCM detected at autopsy for this case. In another case, a pathogenic variant was detected in *LDLR,* NM_000527.5:c.1246C > T. The phenotype of this case was HCM whereas genetic variants in *LDLR* are usually causative of familial hypercholesterolemia (FH) potentially suggesting an incidental finding. However, it cannot be excluded that it affects the development of cardiac hypertrophy.

For the forensic cases (*n* = 12), two VUS with relevance to the phenotypes were detected. A missense variant in *TNNC1*, NM_003280.3:c.435C > A, was called in a case that was referred on the basis of suspected arrhythmia, and where a cardiac disarray pattern was noted at autopsy (see Table 6). Pathogenic variants in *TNNC1* usually cause cardiomyopathy [42]. In another case, where arrhythmia, early repolarisation syndrome (ERS), or Brugada was suspected, a VUS in *CACNB2,* NM_201590.3:c.1717C > T, was detected. Pathogenic variants in this gene have previously been found to be causative of Brugada syndrome [43, 44]. Therefore, this variant may be relevant for the suspected SCD.

The six clinical samples were further analysed by filtering exome variants on the SDGP (166 genes, listed in Supplementary Table 1). In one case, a likely pathogenic variant in *CALM2,* NM_001743.6:c.356A > T, was detected in exon 5. Other variants located in this exon have been reported as pathogenic for LQTS in ClinVar and HGMD [45]. The variant was not present in gnomAD and was predicted pathogenic by bioinformatic prediction tools. We conclude that this variant may be relevant for the SCD. In the other five samples, no variant related to SCD was found, whereas a few VUS were detected (Table 6). In the 12 forensic samples, no pathogenic variant or VUS that could explain the SCD was detected with the SDGP.

## Discussion

In this study, we investigated the extent to which formalin-fixed paraffin-embedded (FFPE) material can be used for whole exome sequencing (WES) with a modified hybridization capture technology from Twist Bioscience. First, 15 SCD cases with matched FFPE tissue and high-quality blood sample, were analysed. Using the results from the blood samples as a reference, the accuracy and predictive values of the WES FFPE procedure were assessed. Second, the method was used in an attempt to find a genetic cause of death in 35 cases of suspected SCD using only FFPE material. A satisfactory high-quality analysis could be performed in 23 of the 35 cases.

Previous publications on molecular autopsies using WES FFPE are sparse. In 2017, Baudhuin et al. first reported the use of WES on FFPE samples in four cases with a clinical phenotype suggesting an inherited cardiovascular disorder [46]. In the same year, Bagnall et al. investigated FFPE samples from five juvenile SCD cases [47]. Lin compared 12 SCD cases using FFPE and corresponding non-formalin fixed samples and showed that all clinically relevant genetic variants identified in the non-fixed samples were confirmed in FFPE samples with variable degree of confidence, but the FFPE samples had an elevated level of false positives and negatives [15]. Our study is, to our knowledge, the largest validation of WES FFPE as a tool for forensic molecular autopsies.

Genetic analysis of post-mortem FFPE samples is associated with difficulties to extract DNA of sufficient quality and quantity, which can be induced by factors such as suboptimal storage conditions and/or delayed formalin fixation. Fixation and embedding protocols vary between laboratories, as may storage conditions. All these factors are difficult to fully control for a genetic laboratory and will likely affect the quantity and quality of the extracted DNA [48]. The extraction method is an additional parameter that may ultimately affect coverage, depth, and variant calling [49, 50].

On the other hand, proper quality control can potentially avoid unnecessary costs of sequencing samples that will not yield sufficient coverage in the end. In this study, quantitative PCR (qPCR), DIN-score, and input DNA average fragment length were assessed for their correlation to library generation and successful sequencing. As illustrated in Fig. 3, the qPCR value is correlated to the % of the exome covered to 20X (*r*^2^ = 0.72) and consequently the sequencing success (in this study arbitrarily defined as % 20X equal to at least 90%). For samples with qPCR ≥ 10, sequencing generally resulted in < 10 M reads and insufficient coverage for confident variant calling. Fragment length and DIN-score did not predict the sequencing success as well as qPCR values but were still weakly correlated to the % of the exome covered to at least 20X (*r*^2^ = 0.23 and *r*^2^ = 0.29 respectively). Iterating the extraction and library preparation using the same FFPE material did not increase the success rate and the fallback is to find new FFPE samples, if available, or other sources for DNA. However, for samples with qPCR < 10 other factors than amplification potential seems to come into play, as these samples result in variable, less predictable, amount of reads. If possible, samples with similar amplification potential should be sequenced together to obtain a more even read distribution between samples, and thereby optimal sample coverage. For poor-quality samples, if there is a sufficient amount of DNA and consumables available for several attempts, we also recommend simultaneous library preparation with and without enzymatic fragmentation, using the same indexes, with the selection of the best library for sequencing after pre-hybridization quality control.

This study compared a set of matched FFPE and high-quality blood samples (*n* = 13) where twice as many raw reads were needed for the former, low-quality, samples to ensure the same level of coverage across all pooled samples. This is in agreement with previous comparative publication [19, 51]. Highly efficient extraction methods is a key to gain sufficient quantity of DNA. The samples analysed in the present study ranged from 1 to 23 years in storage. Other studies have noted that the age of the FFPE material correlates to both quantity and quality [14]. Too few and too young samples were included to draw definite conclusions of the effect of age; however, we do note that successful sequencing was achieved in some of the oldest samples and therefore age alone is not a hinder for attempting molecular autopsy.

When the aim of the genetic analysis is to confirm the presence of a familial variant, we conclude that even very low exome coverage need not be a hinder, as long as the position under investigation is sufficiently covered. Thus, depending on the reason for referral, FFPE material of low quality can be successfully analysed, even if gene panel analysis is not possible, as was indeed observed for one of the samples.

Through comparison of variants from platinum genomes in two Coriell samples (NA12877 and NA12878), with data obtained through processing the same samples using the library preparation protocols for FFPE and blood samples, the concordance of library preparation, sequencing and bioinformatics could be investigated individually. No discernible difference was observed for the two protocols in terms of false positives/negatives (Table 3), suggesting that the protocol itself is not inducing artefacts. A considerable number of false variants were present in both Coriell samples (ranging from 5435 to 5849 variants). Identifying and removing problematic regions in the exome through a stratification strategy as well as only including confident regions greatly reduced the number of false variants (ranging from 209 to 258 variants), see Fig. 6. Calling and interpreting variants in such problematic regions necessitates caution. The remaining false variants constitute a small proportion of the total number of variants (ranging from 0.9 to 1%), and are mostly located in other problematic regions (Supplementary Table 5) but not part of our stratification strategy. We further note that the majority of false negatives were only called by a single caller in the platinum genomes, whereas in contrast, our pipeline use several callers, ultimately only calling variants where there is an agreement in 2 of 3 callers. This approach has been shown to reduce false variants and may in part explain the deviation [52] alluded to above.

The comparison between matched FFPE and blood samples indicates a high raw concordance with average PPV equal to 0.98 and sensitivity equal to 0.97 (Table 5) which is further increased to 0.996 and 0.998 respectively when stratifying for problematic regions (Fig. 7). No distinct artefacts related to formalin-fixation damage could be identified. Our findings are equivalent to a previous exome comparison between matched samples [53]. High concordance and lack of artefacts might in part be explained by the comparatively high and uniform coverage obtained in the FFPE samples. Hybridization capture has previously been shown to yield very uniform sequencing compared to amplicon-based protocols [23, 54].

Estimating the diagnostic yield, i.e. reporting a pathogenic variant connected to the phenotype, is complicated by the inherent uncertainty in variant interpretation, lack of clinical findings which is inevitable for the SCD type of referral, and other factors. A diagnostic yield of 15–30% has been reported for molecular autopsies [8]. The diagnostic yield of WES as a tool for molecular autopsies depends on the analysed cohort, and the availability of family members, with a yield of close to 50% in highly selected cohorts [18]. In this study, three of 24 cases were positive, giving a diagnostic yield of 12.5%.

A heterozygous pathogenic variant in *MYBPC3*, NM_000256.3:c.2490dupT, was detected in our cohort which results in a frameshift, p.(His831fs*2). Loss of function may be due to protein truncation or non-sense mediated decay. Dominant *MYBPC3* and *MYH7* mutations account for the majority of HCM cases [55] and the c.2490dupT variant has been reported in several unrelated individuals with HCM in the literature [46, 55, 56] as well as ClinVar and HGMD. The variant is present in three individuals in the European population of the gnomAD database (where the phenotypes are unclear) corresponding to a frequency of 0.002%. Four family members of the deceased have been screened with one carrier detected, further highlighting the importance of molecular autopsies for living relatives.

A heterozygous pathogenic variant in *KCNQ1*, NM_000218.2:c.573_577del, was detected in a case with suspected arrhythmia with a phenotype implicating the *KCNQ1* gene. Loss of function may result from protein truncation or non-sense mediated decay, which is a known disease mechanism for LQTS/Romano Ward syndrome. This variant has previously been observed in individuals with LQTS [38–40]. It has also been recognized as having a Swedish and Norwegian founder effect [40], but the QT-prolonging effect of heterozygous carriers is variable [41]. The variant is present in four individuals in the European population in gnomAD at 0.003% strengthening the conclusion that the variant is likely to be causative in the present case.

Interestingly, a pathogenic missense variant in *LDLR*, NM_000527.5:c.1246C > T, in a case of HCM was detected. This variant has been reported to be pathogenic causing familial hypercholesterolemia (FH) in various populations [57]. The presence of this variant in the cohort may be an incidental finding, but there is also a possibility that it may have been detrimental. Our past experience shows that pathogenic variants for FH can be found in SCD cases.

In addition to the above variants, three variants of uncertain significance (VUS) and one likely pathogenic variant with relevance to the phenotypes were found. Inclusion of these increases the diagnostic yield to 29%. First, a *CALM2*, NM_001743.6:c.356A > T, missense variant was found in one case. This variant is not present in gnomAD. The base in this position is highly conserved across species, indicating functional significance and bioinformatic prediction tools indicate pathogenicity. The gene *CALM2* has been reported as causative for LQTS, as well as CVPT or LQTS combined with CPVT. The presumed pathogenic variants reported in ClinVar and HGMD are located almost exclusively in exon 5 as is this variant. We therefore assume that the variant is likely to be relevant for SCD; possibly causing LQTS.

Second, a missense VUS in *FBN1,* NM_000138.4:c.1615C > T*,* was detected in a case with a ruptured aorta. The variant, located in exon 13, was not present in gnomAD. In addition, it was located in a highly conserved region of fibrillin-1, where other pathogenic genetic variants have been found in connection to TAAD and Marfan syndrome. Bioinformatic prediction tools indicate that the variant is pathogenic. Despite these pieces of evidence, the variant was classified as VUS but may be relevant for the patient’s disease.

Third, a VUS was detected in *TNNC1*, NM_003280.3:c.435C > A, in a case where a cardiac disarray pattern was noted at autopsy, possibly indicating cardiomyopathy. This variant has previously been reported in the literature in cases of HCM, DCM, and left ventricular non-compaction cardiomyopathy (LVNC) [58–60]. Experimental data suggests a functional change of the altered protein [58, 59] indicating pathogenicity. However, we argue that the experimental evidence is of moderate strength, the variant is present in the European population in gnomAD at 0.02% and therefore its classification is VUS. Nevertheless, we speculate that the variant may well be relevant for the observed cardiac disarray pattern.

Fourth, a VUS was detected in *CACNB2,* NM_201590.3:c.1717C > T*,* with a frequency of only 0.002% amongst Europeans in gnomAD. Variants in *CACNB2* are generally connected to Brugada syndrome, SUD, or arrhythmias. However, the variant is not located in a conserved region, or in the vicinity of other known pathogenic variants and is also predicted benign by bioinformatic prediction tools. Nevertheless, we speculate that this variant may have caused arrhythmia, endoplasmic reticulum stress (ERS), or Brugada syndrome in this case.

Detailed phenotyping in combination with analysis of a few genes with high evidence of gene-disease relationship often has the advantage of a high diagnostic yield with few VUS [18]. Collection and curation of these high-evidence gene-disease relationships are carried out by initiatives like ClinGen [33] and PanelApp [32]. Broader genetic investigations using larger gene panels (> 150 genes) have the advantage of potentially finding pathogenic variants in genes more loosely associated with disease, a possible use of phenotypic data for ranking of variants, but the disadvantage of increasing the numbers of VUS [18, 61, 62]. Furthermore, with broader panels follows the increased risk of finding variants unrelated to the death. We have identified situations that favour the use of broader genetic analyses: (1) in cases where the phenotypic description is lacking, undetailed or summative, and cannot be further exploited; (2) in cases where a heritable cardiac disease may have overlapping phenotypes, like ARVC, HCM, and DCM. Variants in certain genes can give different phenotypic appearance depending on variant location. For example, SCD in HCM is caused mainly by ventricular arrhythmias. Most disease-causing variants in *MYH7* give rise to hypertrophy, while some variants in *MYH7* can also induce atrial fibrillation before hypertrophy appears, mimicking for example LQTS. [63, 64].

There are some limitations to variant detection in our present study. Despite efforts of thorough panel design, there could still be relevant genes missing in our designated panels. As knowledge is growing, new causative genes will emerge and our panels and interpretation may have to be revised. In particular, our study focuses on a set of 166 genes strongly associated with cardiac-related diseases whereas an expanded panel encompassing virtually any number of genes could be generated, but with the caveat of producing an excess of incidental findings. Another limitation of this study is that exome sequencing with short reads, i.e. < 150 bp, does not generally allow for the detection of repeat expansions and therefore some genetic conditions like myotonic dystrophy cannot be detected. However, we argue that genetic variants such as repeat expansions may have limited use in SCD cases. Lastly, our current bioinformatic pipeline is lacking reliable copy number variant (CNV) callers for FFPE samples. Hence, heterozygous CNVs are usually not identified. Updates to our pipeline will include CNV callers for improved diagnostics.

## Conclusion

Despite the challenge of working with poor FFPE material obtained from SCD cases, the modified hybridization capture protocol from Twist Bioscience with UMI followed by Illumina NGS sequencing allowed for successful sequencing in 65% of the included samples. To limit the cost of failed sequencing, qPCR is a valuable method for quality control. We demonstrate that WES of FFPE DNA with qPCR scores > 10 is mostly unsuccessful and resampling from other FFPE material, if available from the case, may be a better option. Optimal sequencing of FFPE samples is obtained by pooling libraries of roughly equal quality in the same sequencing batch, e.g. for instance based on the qPCR values. By comparing variants in matched FFPE tissue and blood samples from the identical origin, we show that the outcome is characterized by high concordance between matched samples (PPV > 0.99 and Sensitivity > 0.99) across the exome. In the successfully sequenced samples (24 out of 35) we assessed the genetic variants using a stepwise filtering approach. A likely pathogenic variant that correlated well with the phenotype of the case was detected in two cases. The presence of one familial variant was confirmed in another case. In addition, in four cases, a VUS relevant to the clinical description of the case was found. Our study therefore provides evidence of the feasibility, accuracy, and usefulness of whole exome sequencing of FFPE material.

## Supplementary Information

Below is the link to the electronic supplementary material.

Supplementary file1 (DOCX 5634 KB)

## Data Availability

The datasets generated during and/or analysed during the current study are available from the corresponding author upon reasonable request.
