# Supplementary material for: Whole exome sequencing of FFPE samples—expanding the horizon of forensic molecular autopsies
Source: Int J Legal Med. 2022 Nov 8;137(4):1215–34. doi: 10.1007/s00414-022-02906-x (PMC10247852; doi:10.1007/s00414-022-02906-x)
Supplement: Supplementary file 1 — Supplementary file1 (DOCX 5634 KB) [file 414_2022_2906_MOESM1_ESM.docx]

# Supplementary data for manuscript entitled: *Whole exome sequencing of FFPE samples*

# *- expanding the horizon of forensic molecular autopsies*

Supplementary Table 1. Content and transcripts for the sudden death gene panel, SDGP, containing 166 genes. Transcripts are obtained from HGMD – Human Gene Mutation Database.

| Sudden Death Gene Panel (SDGP) content | Transcript (HGMD) |
| --- | --- |
| *AARS2* | NM_020745.3 |
| *ABCC6* | NM_001171.5 |
| *ACAD9* | NM_014049.4 |
| *ACADVL* | NM_000018.3 |
| *ACTA1* | NM_001100.3 |
| *AGK* | NM_018238.3 |
| *AGL* | NM_000642.2 |
| *AGPAT2* | NM_006412.3 |
| *ALMS1* | NM_015120.4 |
| *ALPK3* | NM_020778.4 |
| *ANO5* | NM_213599.2 |
| *APOA1* | NM_000039.2 |
| *ATPAF2* | NM_145691.3 |
| *BRAF* | NM_004333.5 |
| *CALM1* | NM_006888.4 |
| *CALM2* | NM_001743.5 |
| *CALM3* | NM_005184.3 |
| *CALR3* | NM_145046.4 |
| *CAPN3* | NM_000070.2 |
| *CASZ1* | NM_001079843.2 |
| *CBL* | NM_005188.3 |
| *CDH2* | NM_001792.4 |
| *CHRM2* | NM_000739.2 |
| *CLCA2* | NM_006536.6 |
| *COX15* | NM_004376.6 |
| *CPT2* | NM_000098.2 |
| *CRPPA* | NM_001101426.3 |
| *CRYAB* | NM_001885.2 |
| *CTNNA3* | NM_013266.3 |
| *DBH* | NM_000787.3 |
| *DNAJC19* | NM_145261.3 |
| *DOLK* | NM_014908.3 |
| *DPM3* | NM_153741.1 |
| *DTNA* | NM_001390.4 |
| *DYSF* | NM_003494.3 |
| *EEF1A2* | NM_001958.3 |
| *ELAC2* | NM_018127.6 |
| *ENPP1* | NM_006208.2 |
| *EPG5* | NM_020964.2 |
| *ETFA* | NM_000126.3 |
| *ETFB* | NM_001985.2 |
| *ETFDH* | NM_004453.3 |
| *FAH* | NM_000137.2 |
| *FBXL4* | NM_012160.4 |
| *FBXO32* | NM_058229.3 |
| *FHOD3* | NM_025135.4 |
| *FKRP* | NM_024301.4 |
| *FKTN* | NM_001079802.1 |
| *FLNC* | NM_001458.4 |
| *FOXD4* | NM_207305.4 |
| *FOXRED1* | NM_017547.3 |
| *FXN* | NM_000144.4 |
| *GAA* | NM_000152.4 |
| *GATA4* | NM_002052.4 |
| *GATA5* | NM_080473.4 |
| *GATA6* | NM_005257.5 |
| *GATAD1* | NM_021167.4 |
| *GATC* | NM_176818.2 |
| *GBE1* | NM_000158.3 |
| *GFM1* | NM_024996.5 |
| *GLB1* | NM_000404.3 |
| *GLRA1* | NM_000171.3 |
| *GMPPB* | NM_013334.3 |
| *GSK3B* | NM_002093.3 |
| *GTPBP3* | NM_133644.3 |
| *GUSB* | NM_000181.3 |
| *HADHA* | NM_000182.4 |
| *HAND1* | NM_004821.2 |
| *HAND2* | NM_021973.2 |
| *HCN4* | NM_005477.2 |
| *HFE* | NM_000410.3 |
| *HRAS* | NM_005343.3 |
| *IDUA* | NM_000203.4 |
| *ILK* | NM_004517.3 |
| *JPH2* | NM_020433.4 |
| *KCNA5* | NM_002234.3 |
| *KLHL24* | NM_017644.3 |
| *KRAS* | NM_004985.4 |
| *LAMA2* | NM_000426.3 |
| *LARGE1* | NM_004737.5 |
| *LEMD2* | NM_181336.3 |
| *LMOD2* | NM_207163.2 |
| *LRRC10* | NM_201550.3 |
| *LZTR1* | NM_006767.3 |
| *MAP2K1* | NM_002755.3 |
| *MAP2K2* | NM_030662.3 |
| *MAP3K8* | NM_005204.3 |
| *MIPEP* | NM_005932.3 |
| *MLYCD* | NM_012213.2 |
| *MRPL3* | NM_007208.3 |
| *MRPL44* | NM_022915.3 |
| *MRPS22* | NM_020191.2 |
| *MTO1* | NM_012123.3 |
| *MYBPHL* | NM_001010985.2 |
| *MYL4* | NM_001002841.1 |
| *MYO18B* | NM_032608.6 |
| *MYOT* | NM_006790.2 |
| *MYPN* | NM_032578.3 |
| *MYRF* | NM_001127392.2 |
| *NDUFAF2* | NM_174889.4 |
| *NF1* | NM_000267.3 |
| *NKX2-5* | NM_004387.3 |
| *NONO* | NM_001145408.1 |
| *NOS1AP* | NM_014697.2 |
| *NRAP* | NM_001261463.1 |
| *NRAS* | NM_002524.4 |
| *NUP155* | NM_153485.2 |
| *PARS2* | NM_152268.3 |
| *PCCA* | NM_000282.3 |
| *PCCB* | NM_000532.4 |
| *PHOX2B* | NM_003924.3 |
| *PLEC* | NM_000445.4 |
| *PLEKHM2* | NM_015164.3 |
| *PNPLA2* | NM_020376.3 |
| *POMT1* | NM_007171.3 |
| *PPA2* | NM_176869.2 |
| *PPCS* | NM_024664.3 |
| *PPP1CB* | NM_206876.1 |
| *PRDM16* | NM_022114.3 |
| *PTPN11* | NM_002834.4 |
| *QRSL1* | NM_018292.4 |
| *RAF1* | NM_002880.3 |
| *RASA2* | NM_006506.3 |
| *RBCK1* | NM_031229.3 |
| *RIT1* | NM_006912.5 |
| *RMND1* | NM_017909.3 |
| *RRAS* | NM_006270.4 |
| *SALL4* | NM_020436.4 |
| *SCN10A* | NM_006514.3 |
| *SCNN1B* | NM_000336.2 |
| *SCNN1G* | NM_001039.3 |
| *SCO1* | NM_004589.3 |
| *SCO2* | NM_005138.2 |
| *SDHA* | NM_004168.3 |
| *SELENON* | NM_020451.2 |
| *SGCA* | NM_000023.3 |
| *SGCB* | NM_000232.4 |
| *SGCD* | NM_000231.2 |
| *SGCG* | NM_000231.2 |
| *SHOC2* | NM_007373.3 |
| *SLC22A5* | NM_003060.3 |
| *SLC25A20* | NM_000387.5 |
| *SLC25A3* | NM_005888.3 |
| *SLC25A4* | NM_001151.3 |
| *SLC4A3* | NM_201574.2 |
| *SMCHD1* | NM_015295.2 |
| *SOS1* | NM_005633.3 |
| *SOS2* | NM_006939.3 |
| *SPEG* | NM_005876.4 |
| *SPRED1* | NM_152594.2 |
| *STAG2* | NM_001042749.2 |
| *TAB2* | NM_015093.5 |
| *TBX20* | NM_001077653.2 |
| *TBX5* | NM_000192.3 |
| *TECRL* | NM_001010874.4 |
| *TMEM70* | NM_017866.5 |
| *TNNI3K* | NM_015978.2 |
| *TOR1AIP1* | NM_001267578.1 |
| *TRDN* | NM_006073.3 |
| *TRIM32* | NM_012210.3 |
| *TRPM4* | NM_017636.3 |
| *TSFM* | NM_001172696.1 |
| *VARS2* | NM_001167734.1 |
| *VCP* | NM_007126.4 |
| *VPS13A* | NM_033305.2 |
| *XK* | NM_021083.3 |

Supplementary Table 2. Content and transcripts for the cardiodiagnostic gene panel, CDGP, containing 84 genes. Transcripts are obtained from HGMD – Human Gene Mutation Database.

| Cardiodiagnostic gene panel (CDGP) content | Transcript (HGMD) |
| --- | --- |
| *ABCC9* | NM_005691.3 |
| *ACTA2* | NM_001613.2 |
| *ACTC1* | NM_005159.4 |
| *ACTN2* | NM_001103.2 |
| *AKAP9* | NM_005751.4 |
| *ANK2* | NM_001148.4 |
| *ANKRD1* | NM_014391.2 |
| *APOB* | NM_000384.2 |
| *BAG3* | NM_004281.3 |
| *CACNA1C* | NM_000719.6 |
| *CACNA2D1* | NM_000722.2 |
| *CACNB2* | NM_201590.2 |
| *CASQ2* | NM_001232.3 |
| *CAV3* | NM_033337.2 |
| *CBS* | NM_001178009.1 |
| *COL3A1* | NM_000090.3 |
| *COL5A1* | NM_000093.3 |
| *COL5A2* | NM_000393.3 |
| *CSRP3* | NM_003476.4 |
| *DES* | NM_001927.3 |
| *DMD* | NM_004006.2 |
| *DSC2* | NM_024422.3 |
| *DSG2* | NM_001943.3 |
| *DSP* | NM_004415.2 |
| *EMD* | NM_000117.2 |
| *FBN1* | NM_000138.4 |
| *FBN2* | NM_001999.3 |
| *FHL1* | NM_001449.4 |
| *FHL2* | NM_201555.1 |
| *GLA* | NM_000169.2 |
| *GPD1L* | NM_015141.3 |
| *JUP* | NM_002230.2 |
| *KCNE1* | NM_000219.4 |
| *KCNE2* | NM_172201.1 |
| *KCNE3* | NM_005472.4 |
| *KCNH2* | NM_000238.3 |
| *KCNJ2* | NM_000891.2 |
| *KCNJ5* | NM_000890.3 |
| *KCNJ8* | NM_004982.3 |
| *KCNQ1* | NM_000218.2 |
| *LAMP2* | NM_002294.2 |
| *LDB3* | NM_001080116.1 |
| *LDLR* | NM_000527.4 |
| *LDLRAP1* | NM_015627.2 |
| *LMNA* | NM_170707.3 |
| *MYBPC3* | NM_000256.3 |
| *MYH11* | NM_002474.2 |
| *MYH6* | NM_002471.3 |
| *MYH7* | NM_000257.2 |
| *MYL2* | NM_000432.3 |
| *MYL3* | NM_000258.2 |
| *MYLK* | NM_053025.3 |
| *MYLK2* | NM_033118.3 |
| *MYOZ2* | NM_016599.4 |
| *NEBL* | NM_006393.2 |
| *NEXN* | NM_144573.3 |
| *PCSK9* | NM_174936.3 |
| *PKP2* | NM_004572.3 |
| *PLN* | NM_002667.3 |
| *PRKAG2* | NM_016203.3 |
| *RBM20* | NM_001134363.1 |
| *RYR2* | NM_001035.2 |
| *SCN1B* | NM_001037.4 |
| *SCN3B* | NM_018400.3 |
| *SCN4B* | NM_174934.3 |
| *SCN5A* | NM_198056.2 |
| *SLC2A10* | NM_030777.3 |
| *SMAD3* | NM_005902.3 |
| *SNTA1* | NM_003098.2 |
| *TAZ* | NM_000116.3 |
| *TCAP* | NM_003673.3 |
| *TGFB2* | NM_001135599.2 |
| *TGFB3* | NM_003239.2 |
| *TGFBR1* | NM_004612.2 |
| *TGFBR2* | NM_003242.5 |
| *TMEM43* | NM_024334.2 |
| *TMPO* | NM_003276.2 |
| *TNNC1* | NM_003280.2 |
| *TNNI3* | NM_000363.4 |
| *TNNT2* | NM_001001430.2 |
| *TPM1* | NM_001018005.1 |
| *TTN* | NM_133378.4 |
| *TTR* | NM_000371.3 |
| *VCL* | NM_014000.2 |

Supplementary Table 3. Content and transcripts for the aorta panel, containing 27 genes. Transcripts are obtained from HGMD – Human Gene Mutation Database.

| Aorta gene panel content | Transcript (HGMD) |
| --- | --- |
| *ACTA2* | NM_001613.2 |
| *ARIH1* | NM_005744.5 |
| *BGN* | NM_001711.6 |
| *COL3A1* | NM_000090.3 |
| *EFEMP2* | NM_016938.5 |
| *ELN* | NM_000501.4 |
| *FBN1* | NM_000138.4 |
| *FBN2* | NM_001999.3 |
| *FLNA* | NM_001456.4 |
| *FOXE3* | NM_012186.3 |
| *HCN4* | NM_005477.3 |
| *LOX* | NM_002317.7 |
| *LTBP3* | NM_001130144.3 |
| *MAT2A* | NM_005911.6 |
| *MFAP5* | NM_003480.4 |
| *MYH11* | NM_002474.3 |
| *MYLK* | NM_053025.3 |
| *NOTCH1* | NM_006258.4 |
| *PRKG1* | NM_006258.4 |
| *SKI* | NM_003036.4 |
| *SLC2A10* | NM_030777.3 |
| *SMAD2* | NM_005901.6 |
| *SMAD3* | NM_005902.3 |
| *SMAD4* | NM_005359.6 |
| *TGFB2* | NM_001135599.2 |
| *TGFB3* | NM_003239.2 |
| *TGFBR1* | NM_004612.2 |
| *TGFBR2* | NM_003242.5 |

Supplementary Table 4. Summary of the FFPE samples included in the current study including average fragment length, degree of fragmentation, DIN score and qPCR values prior to library preparation. The last two columns describe the average library fragment length as well as the sequencing batch. Moderate degree of fragmentation is defined as fragment length > 1000 bp and DIN-score > 3. High degree of fragmentation is defined as fragment length < 1000 bp and DIN-score < 3. Abbreviations: FFPE = formalin-fixed paraffin-embedded.

| Sample | Fragment length (bp) | DIN score | Degree of fragmentation | qPCR | Library fragment length (bp) | Sequence batch |
| --- | --- | --- | --- | --- | --- | --- |
| Sample 1 | 2038 | 2.7 | Moderate | 2.9 | 468 | Batch5 |
| Sample 2 | 2175 | 5.8 | Moderate | 5.7 | 253 | Batch4 |
| Sample 3 | 473 | 1.7 | High | 9.5 | 263 | Batch6 |
| Sample 4 | 1662 | 4 | Moderate | 6.1 | 252 | Batch3 |
| Sample 5 | 1639 | 3.7 | Moderate | 5.6 | 272 | Batch5 |
| Sample 6 | 2304 | 3.8 | Moderate | 2.9 | 281 | Batch5 |
| Sample 7 | 2492 | 5.1 | Moderate | 1.6 | 475 | Batch1 |
| Sample 8 | 266 | 1.5 | High | 6 | 214 | Batch1 |
| Sample 9 | 1614 | 4.6 | Moderate | 5.5 | 282 | Batch3 |
| Sample 10 | 1557 | 3.2 | Moderate | 3.5 | 269 | Batch6 |
| Sample 11 | 1578 | Missing | Moderate | Missing | 305 | Batch3 |
| Sample 12 | 851 | 3 | High | 3.2 | 269 | Batch1 |
| Sample 13 | 470 | 1.5 | High | 3.5 | 255 | Batch1 |
| Sample 14 | 2107 | 2.6 | Moderate | 2.3 | 282 | Batch2 |
| Sample 15 | 2331 | 4.3 | Moderate | 3.6 | 375 | Batch1 |
| Sample 16 | 1770 | 3.4 | Moderate | 4.7 | 266 | Batch5 |
| Sample 17 | 3296 | 5.8 | Moderate | 1.8 | 362 | Batch1 |
| Sample 18 | 747 | 1.5 | High | 5.2 | 272 | Batch4 |
| Sample 19 | 2005 | 3.5 | Moderate | 2.4 | 343 | Batch1 |
| Sample 20 | 2228 | 4.4 | Moderate | 2.3 | 345 | Batch1 |
| Sample 21 | 1880 | 6.1 | Moderate | 3.1 | 285 | Batch5 |
| Sample 22 | 2558 | 4.5 | Moderate | 3 | 287 | Batch2 |
| Sample 23 | 2698 | 3.1 | Moderate | 6.1 | 322 | Batch3 |
| Sample 24 | 2776 | 5 | Moderate | 0.7 | 306 | Batch4 |
| Sample 25 | 891 | 2.2 | High | 14.8 | 173 | Batch3 |
| Sample 26 | 1403 | 1.6 | High | 12.4 | 170 | Batch4 |
| Sample 27 | 1582 | 2.6 | High | 14.4 | 169 | Batch4 |
| Sample 28 | 480 | 1.6 | High | 13.6 | 307 | Batch2 |
| Sample 29 | 1042 | 1.2 | High | 8.7 | 152 | - |
| Sample 30 | 323 | 1.4 | High | 12.5 | 164 | - |
| Sample 31 | 539 | 2 | High | Missing | 152 | - |
| Sample 32 | Missing | Missing | Missing | Missing |  | - |
| Sample 33 | Missing | Failed | Missing | Missing |  | - |
| Sample 34 | 1027 | 2.9 | High | 10.1 | 277 | Batch3 |
| Sample 35 | 510 | 1.9 | High | 12.0 | 233 | Batch3 |

Supplementary Table 5. Summary of false positives variants in two Coriell samples with annotation based on additional stratification files. Each Coriell sample is analyzed using the FFPE workflow as well as the high quality blood protocol. Both are described in the main text. For NA12877, only 231 unique variants are detected in total, whereas for NA12878 only 226 unique variants are detected in total thus indicating a large number of assay specific false positives. Abbreviations: FFPE = formalin-fixed paraffin-embedded.

| Sample | NA12877 (FFPE) | NA12877 (Blood) | NA12878 (FFPE) | NA12878 (Blood) |  |
| --- | --- | --- | --- | --- | --- |
| False positives (total) | 216 | 258 | 223 | 209 |  |
| In GC-rich regions | 92 | 99 | 80 | 78 |  |
| Segmental duplications | 19 | 18 | 42 | 46 |  |
| NGS problematic regions (low stringency) | 11 | 8 | 31 | 32 |  |
| Self chain | 61 | 65 | 52 | 51 |  |
| Union of all stratification files | 112 | 129 | 109 | 107 |  |
| Remaining false positives after stratification | 104 | 129 | 114 | 102 |  |

Supplemental Table 6. Clinical findings in the FFPE samples. Table depicts analytical request, filtering strategy and genetic findings described with transcript, coding position, protein shift, zygosity and pathogenicity according to ACMG guidelines. Finally, the variant is interpreted in the context of case clinical finding. Abbreviations: AD = autosomal dominant, AR= autosomal recessive, ARVC = arrhythmogenic right ventricular cardiomyopathy, CDGP = cardio diagnostic gene panel, ERS = early repolarization syndrome, FFPE = formalin-fixed paraffin embedded, FH = familial hypercholesterolemia, HCM = hypertrophic cardiomyopathy, LQTS = long QT syndrome SCD = sudden cardiac death, SDGP = sudden death gene panel, SQTS = short QT syndrome, VUS = variant of uncertain significance.

| **Sample** | **Cohort** | **Request** | **Analysis strategy** | **Gene** | **Reference sequence** | **Variant DNA** | **Variant Protein** | **Zygosity** | **Hereditary pattern** | **ACMG classification** | **Interpretation in context of case clinical findings** |
| --- | --- | --- | --- | --- | --- | --- | --- | --- | --- | --- | --- |
| Sample1 | Clinical | SCD.  Familial variant in *DLL4* | Familial variant in *DLL4* | *DLL4* | NM_019074.3 | c.1096T>G | p.(Cys366Glu) | Heterozygot | - | - | Familial variant confirmed |
| Sample2 | Clinical | HCM | CDGP | *MYBPC3* | NM_000256.3 | c.2490dupT | p.(His831fs*2) | Heterozygot | AD | Pathogenic | Likely cause of HCM |
| Sample3 | Forensic | Missing | CDGP | *KCNE1* | NM_000219.6 | c.247G>A | p.(Glu83Lys) | Heterozygot | AR/AD | VUS | Uncertain relevance for SCD |
|  |  |  |  | *MYLK* | NM_053025.4 | c.2629G>A | p.(Val877Met) | Heterozygot | AD | VUS | Uncertain relevance for SCD |
|  |  |  | SDGP | *MYPN* | NM_001256267.2 | c.59A>G | p.(Tyr20Cys) | Heterozygot | AR/AD | VUS | Uncertain relevance for SCD |
| Sample4 | Clinical | Analysis of *KCNQ1* | *KCNQ1* | *KCNQ1* | NM_000218.2 | c.573_577del, | p.(Arg192Cysfs*91) | Heterozygot | AD | Pathogenic | Likely cause of SCD |
| Sample5 | Clinical | Arrhythmia, SCD | CDGP | No findings | | | | | | | |
|  |  |  | SDGP | *FOXRED1* | NM_017547.4 | c.277C>G | p.(Arg93Gly) | Heterozygot | AR | VUS | Unlikely relevance for arrhythmia/SCD |
|  |  |  | SDGP | *FLNC* | NM_001458.4 | c.7289C>T | p.(Ala2430Val) | Heterozygot | AD | VUS | Uncertain relevance for arrhythmia/SCD |
| Sample6 | Forensic | Arrhythmia | CDGP | No findings | | | | | | | |
|  |  |  | SDGP | No findings | | | | | | | |
| Sample7 | Forensic | SCD | CDGP | *RBM20* | NM_001134363.3 | c.2282G>A | p.(Arg761Gln) | Heterozygot | AD | VUS | Uncertain relevance for SCD/Structural anomaly of AV-node |
|  |  |  | SDGP | No findings | | | | | | | |
| Sample8 | Forensic | Arrhythmia, palpitations and syncope | CDGP | *AKAP9* | NM_005751.5 | c.4884A>C | p.(Glu1628Asp) | Heterozygot | AD | VUS | Uncertain relevance for SCD |
|  |  |  | SDGP | *GATA6* | NM_005257.6 | c.730G>T | p.(Gly244Cys) | Heterozygot | AD | VUS | Uncertain relevance for SCD |
| Sample9 | Clinical | SCD/  cardiomyopathy | CDGP | *LDB3* | NM_001080114.2 | c.574G>A | p.(Val192Ile) | Heterozygot | AD | VUS | Unlikely cause for SCD/cardiomyopathy |
|  |  |  | SDGP | *SOS1* | NM_005633.3 | c.3904A>G | p.(Ile1302Val) | Heterozygot | AD | VUS | Uncertain relevance for SCD/cardiomyopathy |
| Sample10 | Forensic | SCD, tall stature, possible enlarged heart, structurally normal | CDGP | *SMAD3* | NM_005902.4 | c.931A>G | p.(Ser311Gly) | Heterozygot | AD | VUS | Uncertain relevance for SCD |
| Sample11 | Clinical | SCD | CDGP | No findings | | | | | | | |
|  |  |  | SDGP | *CALM2* | NM_001743.6 | c.356A>T | p.(Asp119Val) | Heterozygot | AD | VUS | Possibly relevant for SCD |
| Sample12 | Forensic | Arrhythmia, ERS, possibly Brugada | CDGP | *CACNB2* | NM_201590.3 | c.1717C>T | p.(Arg573Cys) | Heterozygot | AD | VUS | Possibly relevant for SCD |
|  |  |  |  | *COL5A1* | NM_000093.5 | c.814A>T | p.(Thr272Ser) | Heterozygot | AD | VUS | Uncertain relevance for SCD |
|  |  |  | SDGP | *FLNC* | NM_001127487.2 | c.1898C>T | p.(Thr633Met) | Heterozygot | AD | VUS | Uncertain relevance for SCD |
| Sample13 | Forensic | Arrhythmia | CDGP | No findings | | | | | | | |
|  |  |  | SDGP | No findings | | | | | | | |
| Sample14 | Clinical | Aorta related genes | Aorta panel | No findings | | | | | | | |
| Sample15 | Forensic | SCD, lung infiltration | CDGP | *COL5A1* | NM_000093.5 | c.4121C>T | p.(Thr1374Met) | Heterozygot | AD | VUS | Uncertain relevance for SCD |
|  |  |  | SDGP | No findings | | | | | | | |
| Sample16 | Clinical | Ruptured aorta dissection | Aorta panel | *FBN1* | NM_000138.4 | c.1615C>T | p.(Arg539Trp) | Heterozygot |  | VUS | Possibly relevant for ruptured aorta |
| Sample17 | Forensic | LQTS/SQTS | CDGP | No findings | | | | | | | |
|  |  |  | SCDP | No findings | | | | | | | |
| Sample18 | Forensic | SCD, w/o structural findings | CDGP | *COL5A1* | NM_000093.5 | c.5414C>T | p.(Pro1805Leu) | Heterozygot | AD | VUS |  |
|  |  |  | SCDP | No findings | | | | | | | |
| Sample19 | Forensic | Arrhythmia, cardiac disarray patterns | CDGP | *TNNC1* | NM_003280.3 | c.435C>A | p.(Asp145Glu) | Heterozygot | AD | VUS | Possibly relevant for SCD |
|  |  |  | SCDP | No findings | | | | | | | |
| Sample20 | Forensic | Arrhythmia | CDGP | *ANK2* | NM_001148.6 | c.3577C>T | p.(Arg1193Cys) | Heterozygot | AD | VUS | Uncertain relevance for SCD |
|  |  |  | SDGP | *COX15* | NM_078470.6 | c.131G>A | p.(Ser44Asn) | Compound heterozygot | AR | VUS | Uncertain relevance for SCD |
|  |  |  |  | *COX15* | NM_078470.6 | c.1217G>A | p.(Arg406Gln) | Compound heterozygot | AR | VUS | Uncertain relevance for SCD |
| Sample21 | Clinical | SCD/cardiomyopathy | CDGP | No findings | | | | | | | |
|  |  |  | SDGP | No findings | | | | | | | |
| Sample22 | Forensic | ARVC | CDGP | No findings | | | | | | | |
|  |  |  | SDGP | No findings | | | | | | | |
| Sample23 | Clinical | SCD/  cardiomyopathy | CDGP | No findings | | | | | | | |
|  |  |  | SDGP | No findings | | | | | | | |
| Sample24 | Clinical | HCM | CDGP | *LDLR* | NM_000527.5 | c.1246 C>T | p.(Arg416Trp) | Heterozygot | AD | Pathogenic | Pathogenic for FH, unclear relevance for HCM |
|  |  |  | CDGP | *ANK2* | NM_001148.6 | c.9825T>G | p.(Asp3275Glu) | Heterozygot | AD | VUS | Uncertain relevance for SCD/HCM |
|  |  |  | SDGP | No findings | | | | | | | |


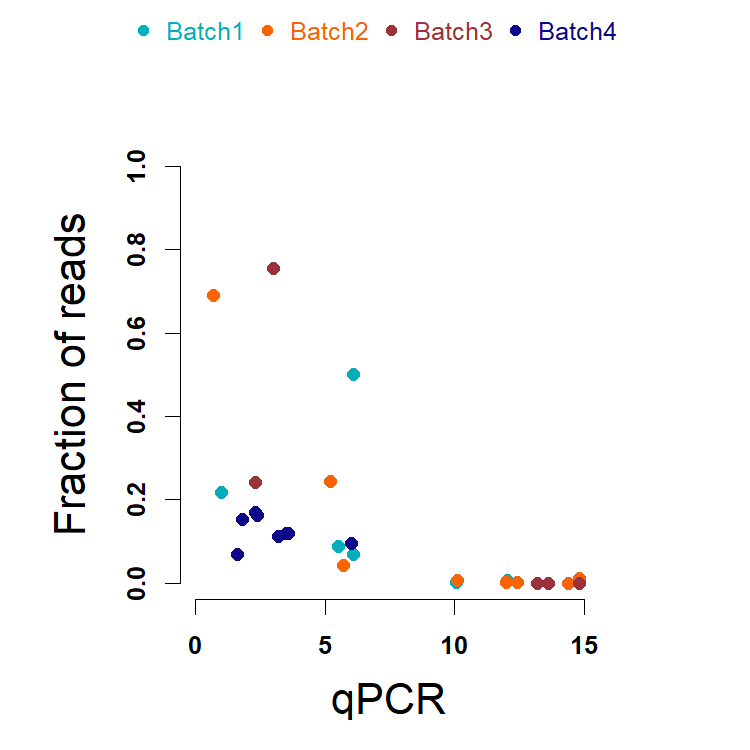


**Supplementary Figure 1. Correlation between qPCR value and the fraction of reads obtained for a batch of sequenced samples (colored according to batch of 8 samples sequenced together in a run). Samples with qPCR value ≥ 10 obtained too few reads to be used for analysis. Samples with a low diversity in qPCR values between samples in the same run demonstrated a more even distribution of reads between the samples (dark blue run), while runs containing samples with very variable qPCR value displayed a pattern where samples with a low qPCR value usually obtain a high number of reads, and samples with a high qPCR value obtain a low number of reads (light blue, orange and red runs)**


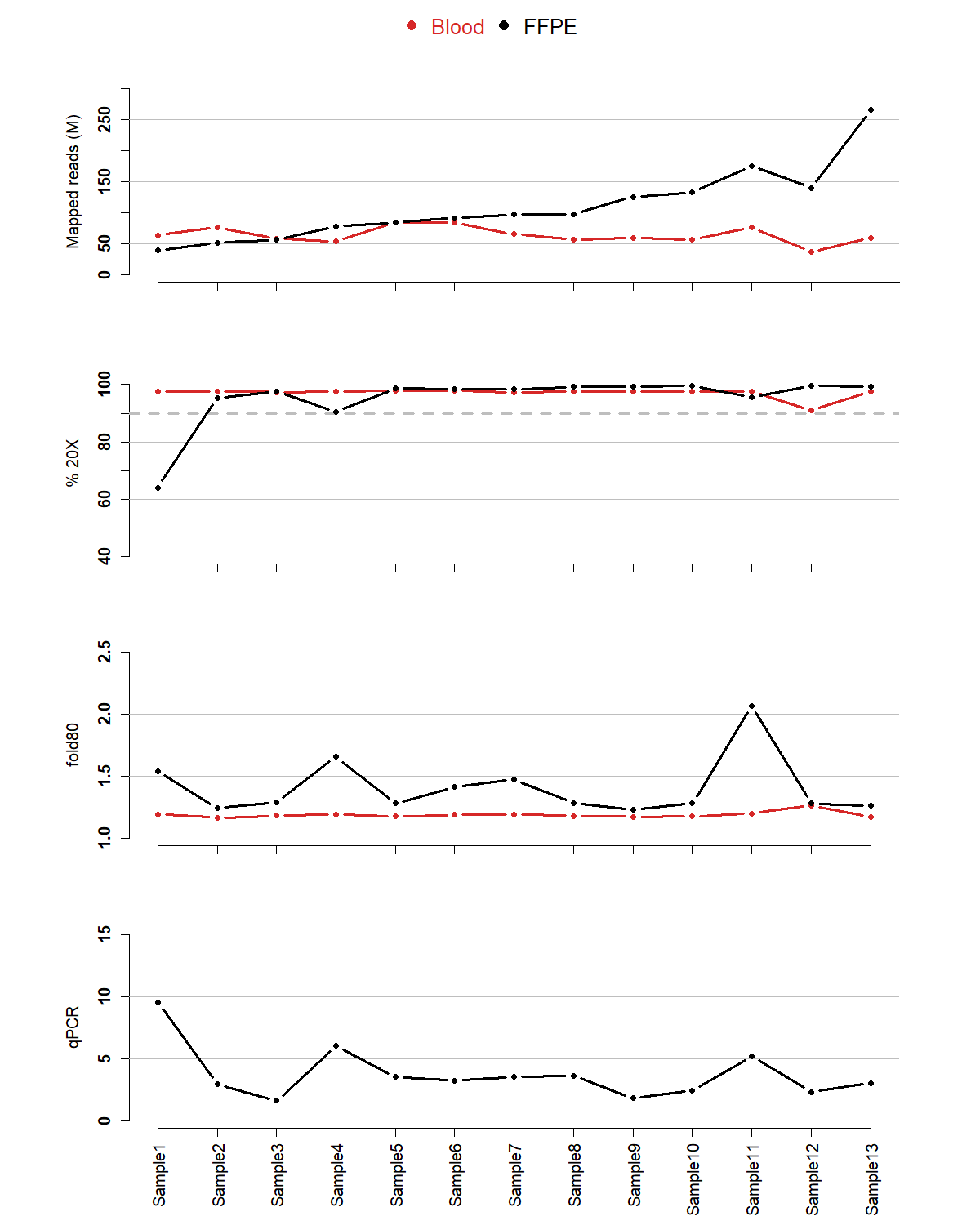


Supplementary Figure 2. Sequencing metrics for the 13 matched FFPE and blood samples (x-axis). From top to bottom, amount of mapped reads to the target BED-file, fraction of bases covered to at least 20X, fold80 penalty and qPCR value.


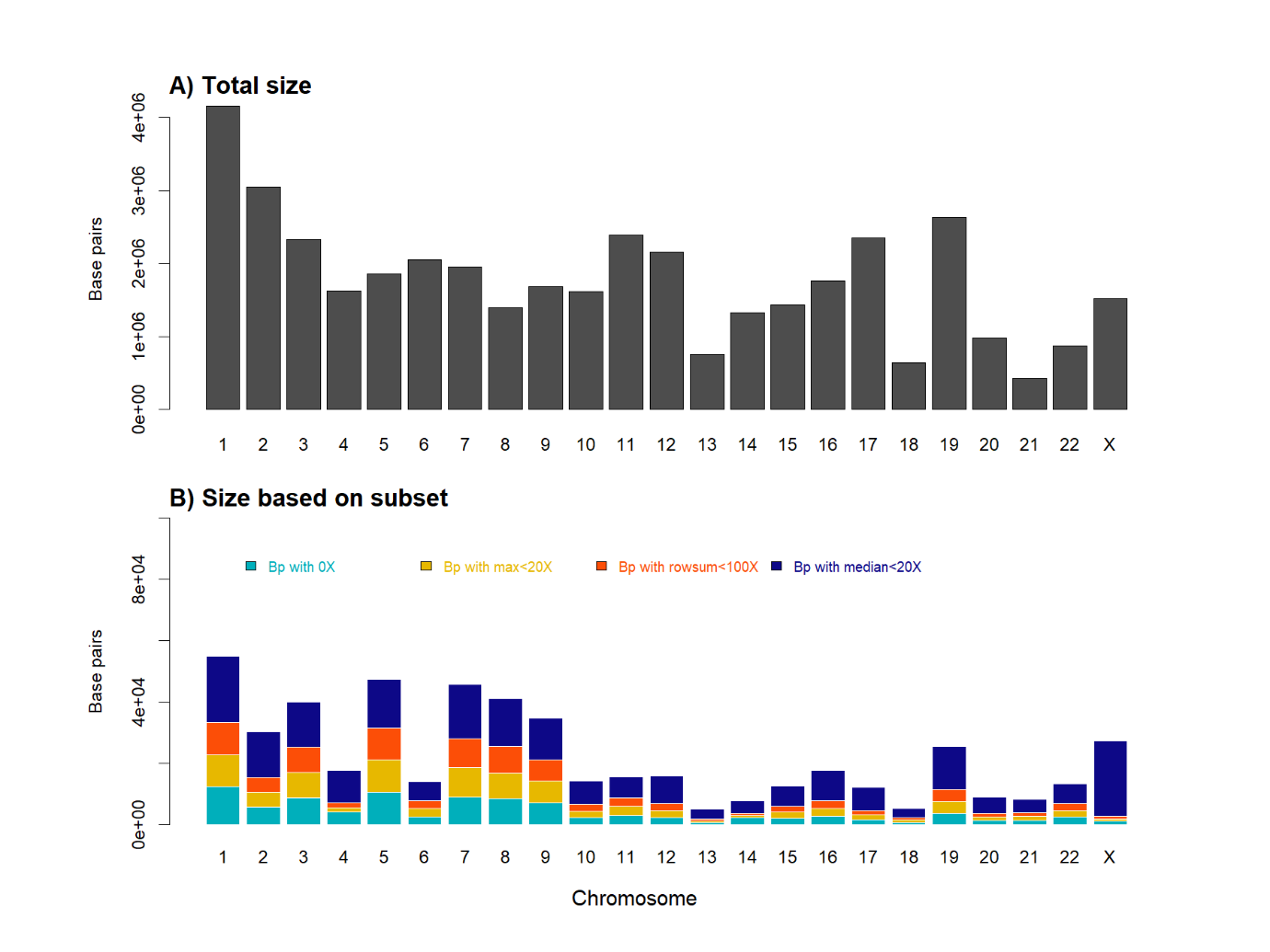


Supplementary Figure 3. Analysis of low coverage target bases using four different approaches, described in detail in the main text. A) Total number of base pairs in the target BED-file across the chromosomes illustrating few exome targets on chr13, chr18 and chr21. B) Stacked bar plot illustrating the number of target bases across the chromosomes for each approach, see legend and main text.


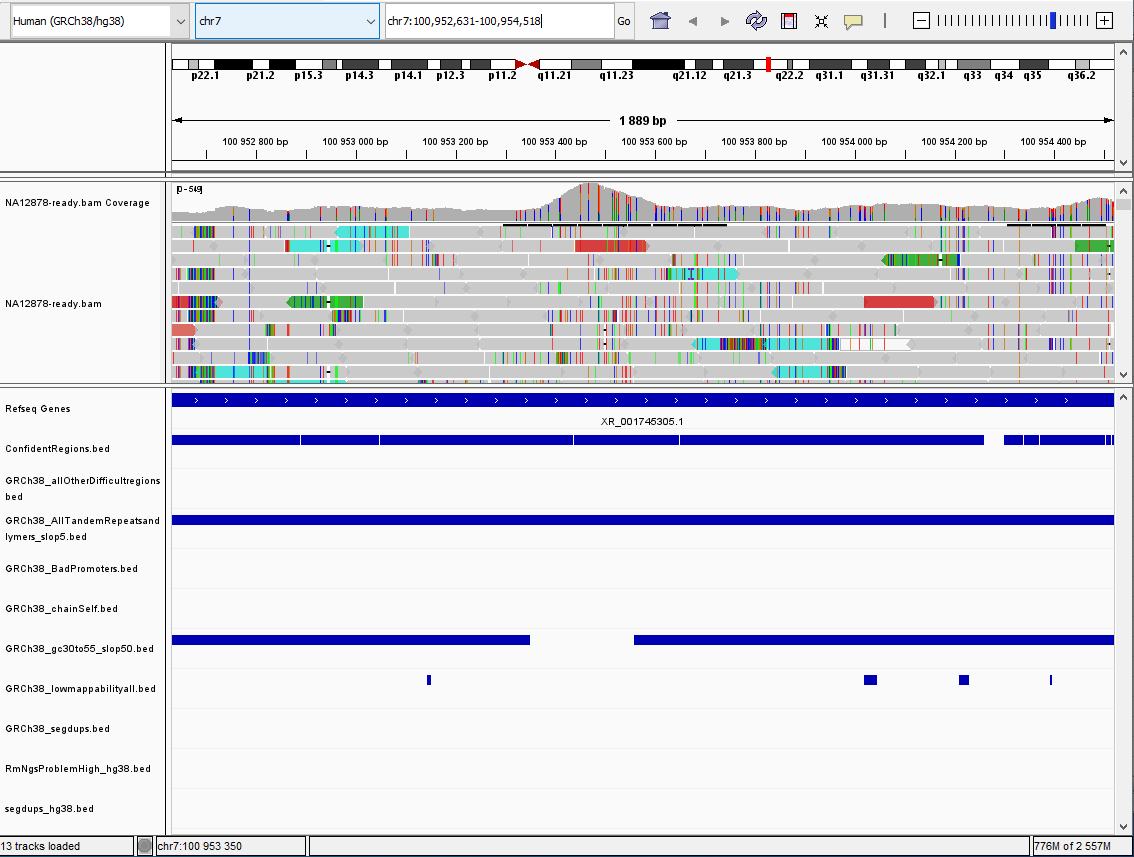


Supplementary Figure 4. Problematic region displayed in IGV for sample NA12878. The coverage is overlayed with BED files containing known problematic regions, in illustration showing that this particular region is enriched with tandem repeats and GC content.
